# A Flexible System-on-Chip Field-Programmable Gate Array Architecture for Prototyping Experimental Global Navigation Satellite System Receivers

**DOI:** 10.3390/s23239483

**Published:** 2023-11-28

**Authors:** Marc Majoral, Carles Fernández-Prades, Javier Arribas

**Affiliations:** Centre Tecnològic de Telecomunicacions de Catalunya (CTTC/CERCA), Parc Mediterrani de la Tecnologia—Building B4, Av. Carl Friedrich Gauss 7, 08860 Castelldefels, Spain; carles.fernandez@cttc.es (C.F.-P.); jarribas@cttc.es (J.A.)

**Keywords:** GNSS, software-defined radio, FPGA, System on Chip, SoC-FPGA

## Abstract

Global navigation satellite system (GNSS) technology is evolving at a rapid pace. The rapid advancement demands rapid prototyping tools to conduct research on new and innovative signals and systems. However, researchers need to deal with the increasing complexity and integration level of GNSS integrated circuits (IC), resulting in limited access to modify or inspect any internal aspect of the receiver. To address these limitations, the authors designed a low-cost System-on-Chip Field-Programmable Gate Array (SoC-FPGA) architecture for prototyping experimental GNSS receivers. The proposed architecture combines the flexibility of software-defined radio (SDR) techniques and the energy efficiency of FPGAs, enabling the development of compact, portable, multi-channel, multi-constellation GNSS receivers for testing novel and non-standard GNSS features with live signals. This paper presents the proposed architecture and design methodology, reviewing the practical application of a spaceborne GNSS receiver and a GNSS rebroadcaster, and introducing the design and initial performance evaluation of a general purpose GNSS receiver serving as a testbed for future research. The receiver is tested, demonstrating the ability of the receiver to acquire and track GNSS signals using static and low Earth orbit (LEO)-scenarios, assessing the observables’ quality and the accuracy of the navigation solutions.

## 1. Introduction

In recent years, GNSS has become an essential part of our daily lives. It is used in many applications requiring accurate positioning, navigation, and timing synchronization. The GNSS industry is evolving at a rapid pace, with new applications emerging that demand complex and customized receiver technology, including the Internet of Things (IoT) [1], augmented reality [2], emergency warning services [3], and intelligent transportation and robotics [4,5,6]. To turn ideas into realistic proofs of concept and to explore the full capability of receivers, researchers require rapid prototyping tools [7]. Prototyping plays a crucial role in research as it allows researchers to test and refine their ideas before full-scale implementation. It facilitates the exploration of concepts, enabling researchers to identify drawbacks and areas for improvement early in the development process. Moreover, it aids in demonstrating the feasibility and potential of a concept, making it easier to attract funding and support for further research.

### 1.1. Motivation

Prototyping with readily available GNSS receivers presents challenges. Presently, GNSS receivers are mainly built-up in application-specific integrated circuits (ASICs), providing low power consumption with small size and low cost but offering limited flexibility [8]. ASICs have limited flexibility for prototyping primarily because they are designed and optimized for specific, dedicated functions or applications. While some commercial devices utilize SDR, they often incorporate proprietary code, restricting reconfigurability through a predefined application programming interface [9].

Prototyping involves practical examination and adjustment of the complete GNSS receiver’s chain, yet proprietary code is typically treated as confidential and inaccessible to researchers. Due to the use of closed software and the increased level of integration and complexity of GNSS ICs, a detailed description of the receiver’s signal processing chain is seldomly available. If the commercial receiver supplies the required measurements, researchers can employ statistical characterization and fitting models in their analysis. Nevertheless, researchers encounter the obstacle of constructing their own receiver in the absence of available measurements. This can be problematic, as receiver prototyping typically falls outside their area of expertise or field of focus [9].

SDR techniques offer a solution to the constrained adaptability of commercial receivers. With its programmable and adaptable nature, SDR technology can be customized precisely to fulfill the user’s specific needs. Moreover, access to inspection and modification of the full GNSS receiver’s signal processing chain can be addressed using free and open-source software (FOSS) [9,10]. FOSS allows users and programmers to edit, inspect, and modify the software’s source code. The open philosophy of FOSS is well suited for education and research, since the source code of the software is available for examination and derivative software can be written without any copyright issues [10]. With FOSS, researchers can freely share and reuse their work, optimizing the development process of applications and increasing productivity in software teams. Code reuse allows developers to focus on the implementation of new features instead of reinventing the wheel for each project. Moreover, code reuse encourages clean, modular, and efficient software, which lowers the likelihood of introducing bugs or inconsistencies.

The main limitations of SDR receivers are power consumption, form factor, and price [8]. The energy usage of embedded devices presents a significant concern. Several SDR receivers rely on software executed by standard processor cores, resulting in excessive power consumption during prolonged battery use, especially when executing intricate algorithms. As an example, the authors tested that GNSS-SDR, a popular software receiver for researchers and developers [11], requires a 28 W 11th gen. Intel Core i7-1185G7 at 3 GHz or similar when running in real-time, in multi-band, multi-constellation mode, and using up to 26 channels. It is thus interesting to investigate SDR architectures that can improve the trade-off between efficiency and flexibility.

The proposed approach is to use SoC-FPGAs, integrating a multi-core embedded processor with FPGA fabric. FPGAs are often more power-efficient than central processing units (CPUs) due to the nature of their architecture and design. Their reconfigurable matrix of logic blocks enables the execution of a diverse array of digital functions, including SDR algorithms. Designed for parallel processing, FPGAs can concurrently manage multiple tasks, enhancing their overall efficiency. This parallelism reduces the overall processing time and decreases the power consumption compared to sequential processing by CPUs. On top of that, FPGAs do not carry the overhead associated with managing complex instruction sets and control logic, which is typical in CPUs. As a result, they can perform specific tasks efficiently with minimal unnecessary operations, leading to lower power consumption.

### 1.2. Contributions

The authors designed a scalable SoC-FPGA architecture for prototyping portable multi-constellation and multi-frequency GNSS receivers using commercial off-the-shelf (COTS) devices. The proposed architecture addresses the flexibility limitations of commercial receivers and enables the implementation of GNSS processing engines using SoC-FPGA platforms, with a power consumption in the range of approximately 5 W to 15 W depending on the complexity of the algorithms. This architecture leverages the flexibility of embedded processors and the massive parallelism and energy efficiency of FPGAs, enabling the implementation of portable receivers that work in real time. The receivers can be implemented on a wide range of SoC-FPGAs featuring various FPGA and embedded processor sizes, focusing on low power consumption, price–performance ratio, or high-capacity and high-performance devices, depending on the target application.

In the proposed architecture, the SoC-FPGA-embedded processor runs GNSS-SDR, a popular and well-known open-source GNSS SDR receiver available online [11,12]. The use of open-source software enables inspection and modification of the GNSS receiver’s chain, facilitating freedom, collaboration, and innovation, and decreasing implementation costs. By default, GNSS-SDR implements a host-based GNSS SDR receiver: a radio-frequency front-end (RFFE) tuned to the GNSS frequency bands receives the satellite signals, performs baseband down-conversion and sampling, and streams the samples to a personal computer (PC) running GNSS-SDR. However, when cross-compiled for execution in embedded processors, GNSS-SDR has the option to offload the most computationally demanding tasks to the FPGA, implementing an SoC-FPGA-based receiver.

The FPGA receives the sample stream coming from the RFFE and implements hardware accelerators for the most computationally expensive algorithms: the acquisition and the tracking processes [13]. The FPGA hardware accelerators are implemented as reusable intellectual property (IP) cores, which can be targeted at many variants of FPGAs.

The authors use a design methodology based on a hardware/software design flow, where FPGA and software development can largely proceed independently. In this way, software can be implemented using a complete FOSS toolchain, independently of the FPGA development tools. Researchers may decide whether to release FPGA IP cores using FOSS or proprietary licenses, as a way to monetize research while also increasing research impact and reputation. Access to the internal aspects of the signal processing algorithms should be possible by inspecting the software version of the FPGA hardware accelerators in GNSS-SDR, together with the documentation provided by the developers of the FPGA IP cores. This approach facilitates code reusability, which is essential for minimizing development time and costs.

This work is a more complete extension of [14,15,16]. The implementation of GNSS receiver hardware accelerators in SoC-FPGA platforms was introduced in [14]. Proof-of-concept implementations in the form of a spaceborne GNSS receiver and a GNSS rebroadcaster were introduced in [15,16]. Therefore, there are parts of this work similar to the prior papers. However, there are many new contributions. The authors explain the proposed architecture in more detail, introducing the proposed design methodology, and a new proof-of-concept demonstrator in the form of a multi-channel, multi-GNSS receiver serving as a testbed for future implementations. The newly introduced prototype is compared against the implementations proposed in [15,16] in terms of hardware resources, size, and power consumption, demonstrating the scalability of the proposed architecture. Finally, the authors conducted new receiver performance measurements, previously unreported, to assess the real-time processing capability of GNSS signals. They also demonstrated the receiver’s ability to obtain navigation solutions using both static and LEO scenarios.

### 1.3. Organization of the Paper

The remainder of this paper is structured as follows. Section 2 reviews previous GNSS SDR receiver implementations in terms of flexibility and energy efficiency. Section 3 describes the proposed SoC-FPGA receiver architecture. Section 4 explains the proposed design methodology to implement experimental algorithms, first focusing on the software and then on the FPGA. Section 5 reviews the proof-of-concept demonstrators implemented using the proposed architecture: the spaceborne GNSS receiver [15] and the GNSS rebroadcaster [16], and the newly introduced general purpose GNSS receiver testbed. Section 6 reports the general purpose receiver performance test results. Finally, Section 7 presents the conclusion and directions for future work.

## 2. Literature Review

Improvements in software and hardware technology have led to a rapid evolution of software GNSS receivers. This is reflected by the increasing number of textbooks and publications on the subject. Textbook [17] focuses on Global Positioning System (GPS) receiver theory and practice, books [18,19] present the advantages of the SDR approach, providing Matlab implementations of a complete GPS receiver. More recently, ref. [20] introduces readers to the Beidou and GPS dual-system software receiver algorithms, and [21] explains how to build and operate multi-GNSS and multi-frequency software receivers with state-of-the-art techniques. Tutorials and descriptions of software GNSS receiver architectures can be found in several publications [22,23,24].

Many publications report the design, implementation, and preliminary performance assessment of GNSS SDR receivers. These systems differ in the purpose of their use and in the technology they are built with. Many devices are implemented in pure software (no programmable logic used), running on a PC or other types of computers [11,25,26,27,28,29,30]. Some software receivers take advantage of the parallel computing performance of graphics processing units (GPUs) [31,32]. Software libraries are also available for the implementation of software GNSS receivers [33]. In general, these systems have a high degree of flexibility and scalability; however, pure software implementations are not usually energy efficient. GNSS receivers implementing computationally expensive algorithms may not be suited for battery-powered embedded devices.

Some works are devoted to the development of FPGA-based platforms for the fast prototyping of GNSS receiver algorithms [34,35,36,37,38], or platforms combining FPGAs and digital signal processors (DSPs) [39]. These platforms have a high degree of flexibility, but they are usually implemented using devices of considerable size and power consumption that are not portable and are not suitable for battery-powered applications. In contrast to this, some publications propose FPGA-based designs targeted at very specific applications such as GNSS in space ([40,41]), GNSS receivers for safety-of-life [42], multi-antenna GNSS receivers [43], and ASIC design [44], among others. In general, these implementations are highly optimized for the application at hand, but they are not usually designed as a general-purpose platform for testing and prototyping non-standard GNSS receiver algorithms.

Some publications deal with the efficiency-flexibility trade-off by exploiting multi-core parallelism to reduce power consumption: A multi-core GNSS baseband processing architecture with many processor cores running at a reduced clock frequency is proposed in [45], and a multi-core architecture with FPGA assistance is proposed in [46], where the processing cores are implemented in FPGA logic. The use of processor cores implemented in the FPGA logic (soft processor cores) has the advantage that the processor cores are fully customizable, and they only include the essential features. However, soft processor cores are slower and less efficient than processor cores implemented in silicon (hard processor cores), such as the multi-processor cores found in SoC-FPGAs. An open computing language (OpenCL)-based implementation is also proposed to balance flexibility and efficiency, where various signal processing blocks are mapped to different hardware resources (PC, GPU, FPGA) [47].

More recently, various publications have proposed SoC-FPGA-based designs for specific applications, combining the massive parallelism and energy efficiency of FPGAs with the flexibility of the embedded processors [48,49,50]. In these cases, the most computationally demanding operations are off-loaded to the FPGA, while the SoC processor is mainly dedicated to compute the GNSS basic measurements and navigation solutions: an SoC-FPGA-based receiver is tested to acquire and track GPS L1 C/A satellites in [48], using recorded signals. A dual-frequency receiver is proposed in [49] to test a direct aid to track the GPS L2 and L5 bands after acquiring the L1 C/A signals. Finally, a receiver for reflectometry applications is proposed in [50].

The materials referenced are applied across platforms including CPUs, GPUs, FPGAs, DSPs, and SoC-FPGAs, either separately or in combination. Several CPU-based devices operate on PCs, while others function on embedded processors. Devices that combine CPUs and FPGAs also operate on personal computers or embedded processors, with certain instances employing soft processors implemented via FPGA logic. Table 1 shows a compilation of the cited materials organized according to their underlying processing units and the date of publication. The compilation was based on a basic search for the most relevant publications. While the earliest implementations were based on software operating on CPUs, an increasing trend is observed towards the use of heterogeneous platforms such as SoC-FPGAS, combining different types of processors and co-processors in the same system. The use of heterogeneous platforms optimizes system performance by assigning specific tasks to the most suitable processing units.

**Table 1:** Table comparing related work based on publication date and underlying processing units.

Year	CPU	CPU, GPU	FPGA, DSP	FPGA	CPU, FPGA	CPU, FPGA, GPU	SoC- FPGA
1997	[25]						
2003	[26]						
2004	[33]				[34]		
2006			[39]				
2008		[31]					
2009	[27]						
2010	[28]	[32]	[40]				
2011	[11]				[41,44,45]		
2012			[42,43]				
2013					[46]		
2014					[35,36]		
2015	[29]						
2018	[30]						
2019					[37]		[48]
2020						[47]	
2021							[49]
2022				[38]			[50]

The research outlined in this paper provides a combination of features not found in previous publications. We propose a flexible SoC-FPGA-based architecture aiming to enhance the balance between flexibility and energy efficiency. While certain referenced works use SoC-FPGAs for specific applications, we introduce a generic design methodology for the development of portable, experimental GNSS receivers, intended for research purposes that demand non-standard features. The proposed methodology facilitates code reuse and allows for the development of GNSS receivers across a range of research applications. In the proposed architecture, the embedded processor operates a popular open-source GNSS software, facilitating code reuse. In this way researchers can save time by avoiding repetitive problem-solving and focus immediately on developing the specific features they require. The software running in the embedded processor is portable to various processor architectures, including the Intel x86-64 [51] and the ARM architectures [52]. Therefore, novel algorithms can be first tested in software, followed by an implementation in the embedded processor. Our work includes the development of three GNSS receiver prototypes and an initial evaluation of their performance using live GNSS signals.

## 3. System Design

### 3.1. Overview

The proposed architecture is based on the use of SoC-FPGAs for the implementation of GNSS SDR receivers. An SoC-FPGA is an integrated circuit that combines the functionalities of both an FPGA and an embedded processor.

An FPGA is a type of programmable, multi-purpose digital chip based on a matrix of configurable logic blocks (CLBs) connected through programmable interconnects. The flexibility of FPGAs allows for programming and reconfiguration anytime, anywhere. The logic blocks contain lookup tables, flip-flops, and a switch matrix, which can be used to implement logic functions and registers. FPGAs also contain special purpose components: memory blocks for dense memory requirements and DSP units for high-speed arthimetic [53,54]. Combining SDR techniques with FPGA technology allows the creation of adaptable signal processing systems. SDR techniques facilitate the processing of radio signals through software algorithms, and FPGAs deliver the essential hardware acceleration to execute these algorithms in real time. FPGAs achieve parallelism by executing various tasks concurrently through the utilization of diverse configurable logic blocks, leading to improved system performance and reduced processing time. The flexibility and reprogrammability of FPGAs enables rapid reconfiguration of the SDR algorithms.

**Figure 1:**
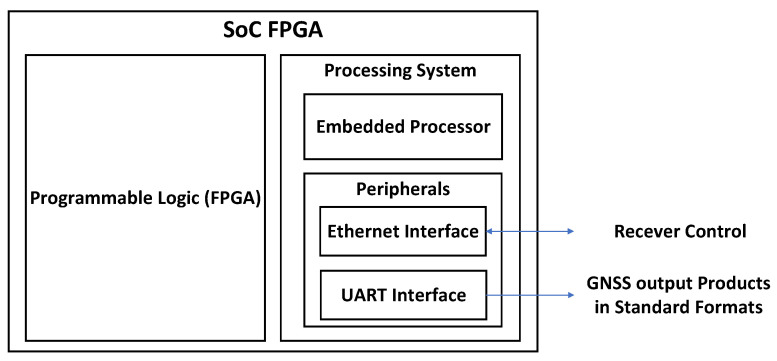
SoC-FPGA block diagram.

Figure 1 illustrates a block diagram of an SoC-FPGA. An SoC-FPGA contains a processing system (PS) and programmable logic (PL). The PS contains an embedded processor and interfaces for connecting to devices outside the SoC-FPGA, such as a universal asynchronous receiver transmitter (UART), or Ethernet. The PL contains FPGA logic tightly coupled with the embedded processor. The SoC-FPGA connects to a double data rate (DDR3) or DDR4 memory for program execution in the embedded processor (the DDR memory is not illustrated in Figure 1).

**Figure 2:**
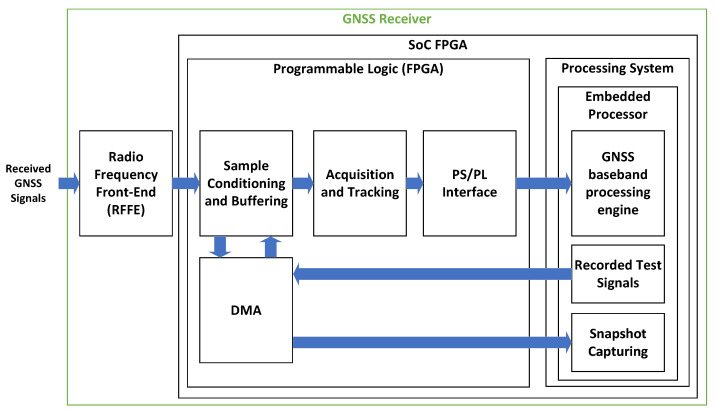
Receiver architecture.

**Figure 3:**
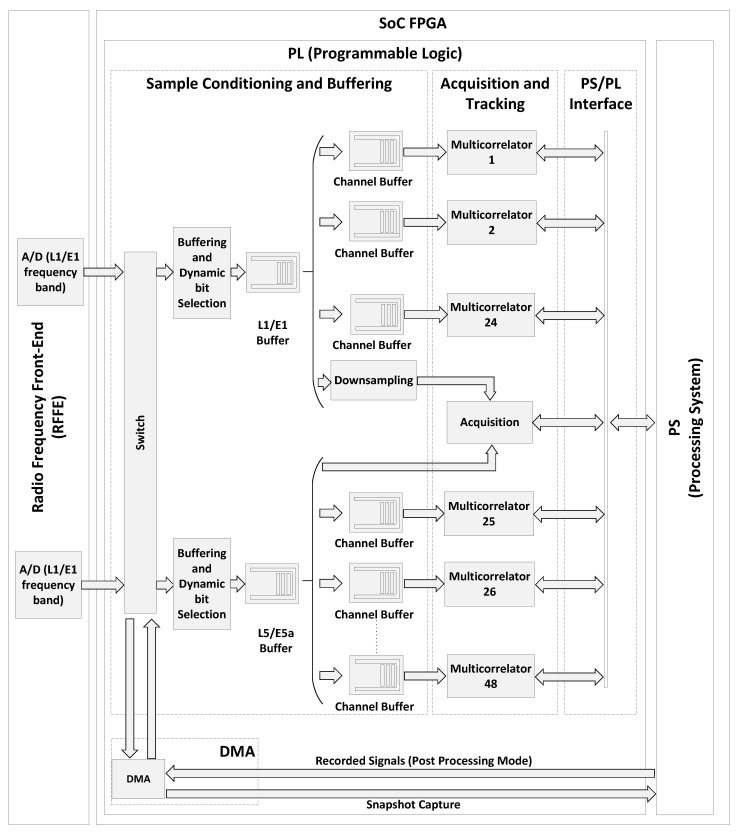
Detailed FPGA design.

**Figure 4:**
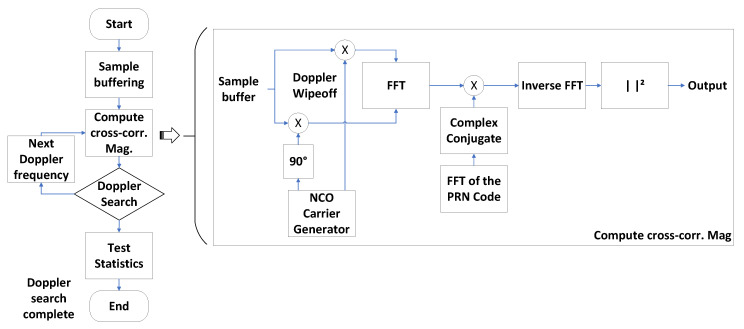
FPGA acquisition hardware accelerator.

**Figure 5:**
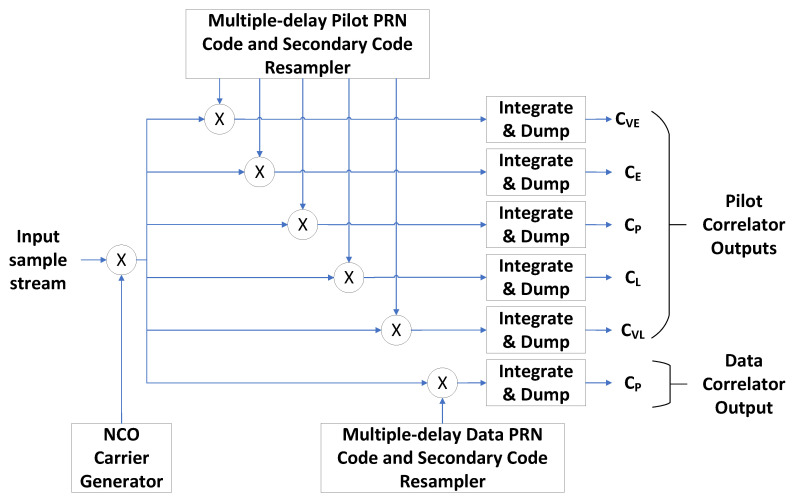
FPGA tracking multicorrelator hardware accelerator.


In the proposed architecture, the SoC-FPGA implements a GNSS receiver. An Ethernet interface in the SoC-FPGA is used to communicate and remotely control the GNSS receiver. Additionally, this Ethernet interface enables the receiver to transmit captured signal snapshots to a remote computer. Furthermore, the receiver can utilize both the Ethernet and UART interfaces to transmit GNSS output products in standard formats to remote devices. These formats include Receiver-Independent Exchange Format Version (RINEX) Files, Radio Technical Commission for Maritime Services (RTCM) messages with configurable rates, and National Marine Electronics Association (NMEA-0183) messages for sensor integration. A detailed description of SoC-FPGAs, providing more details on the elements shown in Figure 1, can be found in [53,54,55]. The proposed GNSS receiver architecture is shown in Figure 2, while the FPGA design is illustrated in more detail in Figure 3. Detailed block diagrams of the acquisition and tracking FPGA hardware accelerators are shown in Figure 4 and Figure 5 respectively.

Figure 2 shows a block diagram of the GNSS receiver architecture. The diagram highlights the main components of the GNSS receiver: the RFFE and the SoC-FPGA. The illustration also depicts the FPGA logic and the Embedded Processor, alongside the primary functional blocks of the GNSS receiver within both components.

The RFFE in Figure 2 is tuned to the desired GNSS frequency bands, performs direct RF to baseband conversion, and digitizes the received signals. Utilizing multi-frequency RFFEs enables the implementation of GNSS receivers that support multiple frequencies.

The FPGA Logic comprises four functional blocks, as illustrated in Figure 2: sample conditioning and buffering, acquisition and tracking, DMA, and PS/PL interface. These blocks are explained below:

1. Sample Conditioning and Buffering: The sample conditioning and buffering block receives the samples coming from the RFFE and implements sample buffering and clock conversion between the front-end interface and the FPGA hardware accelerators. The sample conditioning and buffering block also implements a bit selector, used to dynamically requantize the GNSS signals to map the dynamic range of the incoming samples to the dynamic range of the acquisition and tracking hardware multicorrelators.
2. Acquisition and Tracking: The FPGA incorporates hardware accelerators for the algorithms with the highest computational cost: the acquisition and tracking multicorrelators [13]. The most computationally expensive algorithms are the signal processing stages that process the digitized signals at the sampling rate. However, it is also possible to offload any other processor intensive algorithms to the FPGA.
3. DMA: the FPGA implements a bi-directional direct memory access (DMA). The DMA can be used to run the receiver in post-processing mode using recorded GNSS files, to record the received GNSS signals into files, to capture snapshots, or to send the received GNSS signals to an external device in real time.
4. PS/PL Interface: Interface between the FPGA and the processing system. This interface is implemented using the Advanced Microcontroller Bus Architecture (AMBA) Advanced eXtensible Interface (AXI4) memory-mapped bus. Most SoC-FPGAs support the AMBA architecture. The GNSS baseband engine controls the execution of the acquisition and tracking multicorrelators using a set of memory-mapped registers, and a set of interrupts going from the FPGA to the processing system.

The processor shown in Figure 2 runs GNSS-SDR on a GNU/Linux operating system (OS). GNSS-SDR implements the baseband GNSS processing engine. GNSS-SDR has an option to offload the most computationally demanding tasks to the FPGA. This option can be used when cross-compiled for execution in embedded processors, enabling the execution of the GNSS-SDR in real-time using portable devices.

When working in post-processing mode, the receiver processes recorded GNSS signals. The recorded GNSS signals are usually stored in a non-volatile device such as Secure Digital (SD) card. The embedded processor transfers the recorded signals from the SD card to the system’s DDR memory, and programs the DMA in the FPGA to simultaneously transfer the signals from memory to the hardware accelerators in the FPGA. It would be more efficient to use only the FPGA to transfer the recorded signals directly from the SD card to the hardware accelerators, but that would result in a more complex implementation. The OS running in the embedded processor has proper drivers to enable easy access to external system components such as the SD card, facilitating the use of the embedded processor for transferring the samples from the SD card to the system’s memory. For this reason, the current implementation uses both the DMA and the embedded processor to read the recorded signals.

The process of capturing snapshots also involves the use of DMA and the embedded processor. The utilization of the embedded processor leverages the OS Transmission Control Protocol/Internet Protocol (TCP/IP) software stack, facilitating the utilization of the Ethernet interface for sending the captured snapshots from the system’s memory to a remote computer (Snapshot Capturing in Figure 2). The sample transfer fully occupies the processor, rendering simultaneous real-time operation with GNSS-SDR unfeasible during the transfer.

The proposed architecture has been implemented and tested using the Advanced Micro Devices (AMD)’s Zynq 7000 All Programmable SoC [56] and the Zynq Ultrascale+ Multi Processor SoC (MPSoC) [55] families, demonstrating the flexibility and the scalability of the design. Various types of GNSS receivers can be implemented on a wide range of SoC-FPGAs, starting from a Zynq-7000 SoCs featuring a single-core Cortex A9 ARM processor, 23 k logic cells and 66 DSP slices [57], and up to a Zynq Ultrascale+ MPSoCs with quad-core ARM Cortex-A53 processors, 1143 k logic cells and 2520 DSP slices [58]. The scalability of the Zynq-7000 and the Zynq Ultrascale+ devices enables the implementation of a wide range of GNSS receivers, starting from low-power and small form-factor single-frequency and single-constellation devices and up to multi-frequency, multi-constellation, high-performance receivers implementing highly complex algorithms.

The subsections below provide a more detailed description of the FPGA architecture, the acquisition and tracking multicorrelator hardware accelerators, and the software design.

### 3.2. FPGA Architecture

Figure 3 shows a schematic representation of the FPGA design, providing a comprehensive view of the functional blocks outlined in Figure 2: the sample conditioning and buffering, the acquisition and tracking, the PS/PL interface, and the DMA. The block diagram in Figure 3 showcases the implementation of a multi-frequency multi-constellation GNSS receiver capable of processing GPS L1 C/A, Galileo E1b/c, GPS L5 and Galileo E5a signals.

In Figure 3, a dual-channel RFFE is tuned to the L1/E1 and L5/E5a frequency bands. The RFFE performs RF to baseband conversion, digitizes the received signal with the analog-to-digital (A/D) converters, and sends the digital samples to the FPGA. The FPGA performs signal conditioning and buffering of the received samples. A separate sample buffer is used for each frequency band (labeled as L1/E1 Buffer, and L5/E5a Buffer). The sample buffers are followed by several multicorrelator hardware accelerators. Each tracking multicorrelator is preceeded by a small sample buffer (labeled as Channel Buffer). In this example, the GNSS receiver implements 24 multicorrelator hardware accelerators for each frequency band, and therefore the receiver can potentially track up to 48 GNSS signals simultaneously.

The L1/E1 and the L5/E5a buffers also deliver the received samples to the acquisition hardware accelerator. The input of the acquisition hardware accelerator in the L1/E1 band is preceded by a downsampling filter. By placing the downsampling filter in front of the acquisition, the receiver employs a reduced sampling frequency for the acquisition of the GNSS signals in the E1/L1 frequency band, and subsequently, a higher sampling frequency for tracking the same signals. This arrangement aligns with the fact that the signal detection is not significantly benefited from operating within a bandwidth exceeding that necessary to capture the main lobe of the received signals, which is approximately 2 MHz for GPS L1 C/A signals and 4 MHz for Galileo E1b/c signals, due to the increased noise in the captured signal [59]. However, using a large bandwidth for tracking GNSS signals facilitates a more precise determination of the carrier and code phases of the received signals. Using a reduced sampling frequency for the acquisition of GNSS signals in the E1/L1 frequency band also speeds up the acquisition process.

The FPGA also implements the bi-directional DMA engine. The DMA engine can be used to transfer recorded GNSS signals from the processing system memory to the L1/E1 and the L5/E5a buffers and in this way run the receiver in post-processing mode. The DMA can also be used to capture snapshots from the RFFE and transfer them to the processing system’s memory.

The PS/PL interface in Figure 3 implements an efficient mechanism to enable, disable, configure and exchange information between the processing system and the hardware accelerators in the FPGA. Each hardware accelerator presents a series of accessible read/write registers via an AXI4 slave bus connection with the processing system. In order to reduce the computational load, individual interrupt signals are directed from each hardware accelerator to the processing system. The interrupts trigger acquisition and tracking channels callbacks in the processing system in order to read the results and to reload the acquisition and tracking parameters for processing the next batch of samples. The processing system controls the tracking loops, cycling through the following steps:

1. The software running in the processing system configures the multicorrelator hardware accelerators with updated parameters: coherent integration time, Doppler frequency correction, etc.
2. The multicorrelator hardware accelerator captures a new batch of samples and processes the samples on the fly.
3. The multicorrelator hardware accelerator finishes processing the received samples.
4. The multicorrelator hardware accelerator interrupts the processing system and waits for the multicorrelation results to be read. The software running in the processing system reads those results and operates the tracking loop.

The sample buffers are needed to temporarily store the incoming samples when the multicorrelator hardware accelerators are waiting for the action of the processing system. The processing system runs GNU/Linux. In this way, GNSS-SDR can be easily cross-compiled and used in the embedded platform. However, GNU/Linux is not a real-time operating system. For this reason, the processing system may not immediately react to the interrupts coming from the tracking multicorrelators. When the processing system does not promptly respond to the interrupts, the multicorrelators that is in a waiting state block the flow of samples, exerting back pressure on the channel buffers. During normal operation, when processing GNSS signals in real time, the channel buffers are never completely filled in. However, should a temporary CPU overload occur and result in channel buffer overflow, the L1/E1 and the L5/E5a buffers will store the incoming samples, reducing the probability of sample loss. The L1/E1 buffer and the L5/E5a buffer are identical. Having a separate buffer for each frequency band reduces the probability of sample loss when a temporary channel buffer overflow occurs, as the channel buffer temporarily obstructs the flow of samples in one frequency band only.

The FPGA hardware accelerators are implemented as reusable plug-and-play IP cores enabling portability across many AMD Zynq 7000 All Programmable SoC [56] and Zynq Ultrascale+ MPSoC [55] variants.

The subsections below explain the acquisition and tracking multicorrelator hardware accelerators in more detail.

#### 3.2.1. Acquisition Hardware Accelerator

The purpose of the acquisition hardware accelerator is to detect GNSS signals coming from the visible satellites and to estimate the code phase and the carrier frequency of received signals. The acquisition implements a parallel code phase search algorithm (PCPS) [19]. The PCPS algorithm performs a step-by-step search for the Doppler frequency, while concurrently parallelizing the code phase search, resulting in reduced signal acquisition time and enhanced GNSS receiver performance. Table 2 serves as a guide for the notation and definitions utilized in describing the FPGA implementation of the PCPS acquisition algorithm.

**Table 2:** Notation table for the acquisition algorithm description.

Variable	Definition
$f_{min}$	Minimum tested Doppler frequency
$f_{max}$	Maximum tested Doppler frequency
$\left[f_{min},\;f_{max}\right]$	Doppler frequency span
$f_{D}$	Tested Doppler frequency
$f_{step}$	Doppler search step
$x_{IN}\left[n\right]$	Received GNSS signal input sample stream
$T_{s}$	Sampling period
D[K]	Fast Fourier transform (FFT) of the pseudo-random noise (PRN) code
$|R_{xd}\left(f,\tau \right)|$	Cross-ambiguity function (CAF)

The implementation of the PCPS algorithm is shown in Figure 4 and described in Algorithm 1. The first step of the acquisition algorithm is to buffer the received samples. Then, for each tested Doppler frequency, the acquisition performs the Doppler wipe-off using a numerically controlled oscillator (NCO) carrier generator, and computes the magnitude of the circular cross-correlation between the received signal and a local replica of the satellite’s pseudo-random noise (PRN) code. The circular cross-correlation is computed in the frequency domain. At the end of this process, the acquisition obtains the cross-ambiguity function (CAF). The CAF is a two-dimensional function of the code delay and Doppler frequency. The presence of a signal results in a significant peak detected in the CAF. The acquisition uses a first peak vs. second peak statistic to assert the presence or absence of a satellite signal [19].

The acquisition hardware accelerator runs the complete acquisition algorithm (Algorithm 1) with no processor intervention. The FPGA uses a power-of-two fast Fourier transform (FFT), with a configurable FFT size. The acquisition hardware accelerator performs zero padding when the combination of sampling frequency and length of the PRN codes results in the need to compute a fast Fourier transform (FFT) that is not a power of two. The result is equivalent to using an arbitrary length discrete Fourier transform (DFT), but using a more efficient calculation. The CAF computation is carried out in parallel to the search for the peak value. The acquisition hardware accelerator detects GPS L1 C/A, Galileo E1b+c, GPS L5, and Galileo E5a signals, and it can acquire either the data or the pilot components of the GNSS signals. The current implementation of the acquisition can be configured to use two or four bits per sample.
**Algorithm 1** Acquisition hardware accelerator1:Buffer the received samples2:for $f_{D}=f_{min}$ to $f_{D}=f_{max}$ in $f_{step}$3:   Perform Doppler wipe-off: $x\left[n\right]=x_{IN}\left[n\right]\;\cdot \;e^{-j2\pi f_{D}nT_{s}}$, for n=0,…,N−14:   Compute $X\left[K\right]=FFT_{N}\left(x\left[n\right]\right)$5:   Compute Y[K]=X[K]·D[K], for k=0,…,N−16:   Compute $R_{xd}\left(f_{D},\tau \right)=\frac{1}{N^{2}}IFFT_{N}\left(Y\left[k\right]\right)$7:end for8:Search the peak value and its indices in the search grid: $\left\{S_{max},f_{i},\tau_{j}\right\}=max_{f,\tau }{\left|R_{xd}\left(f,\tau \right)\right|}^{2}$9:Search the second peak value $S_{max_{2}}$ in the same frequency bin of the highest peak $|R_{xd}\left(f_{i},\tau \right){|}^{2}$, for τ=0,…,N−110:Compute test statistic $\Gamma =\frac{S_{max}}{S_{max_{2}}}$11:Compare with threshold value: If Γ>γ12:   Declare positive acquisition and provide $f_{D_{acq}}=f_{i}$ and $\tau_{acq}=\tau_{j}$13:else14:   Declare negative acquisition15:end if

#### 3.2.2. Tracking Multicorrelator Hardware Accelerators

The tracking multicorrelator hardware accelerator computes the correlation of the incoming signal with various local replicas of the satellite’s PRN code, $R_{xd}\left(\tau \right)$. The FPGA also wipes off the PRN secondary code. The software implements the tracking loop, which uses the correlation results obtained in the FPGA to synchronize the local replicas of the PRN code to the incoming signal and to follow the evolution of the signal synchronization parameters as accurately as possible. Table 3 serves as a guide for the notation and definitions utilized in describing the FPGA implementation of the tracking multicorrelator algorithm.

**Table 3:** Notation table for the tracking multicorrelator algorithm description.

Variable	Definition
*N*	Number of samples indicating coherent integration time.
$f_{D}$	Estimated Doppler frequency
$x_{IN}\left[n\right]$	Received GNSS signal input sample stream
$T_{s}$	Sampling period
c[n]	PRN code
s[n]	Secondary code
$R_{xd}\left(\tau \right)$	Correlation of the incoming signal with the PRN code
$C_{VE}$	Very Early correlator output
$C_{E}$	Early correlator output
$C_{P}$	Prompt correlator output
$C_{L}$	Late correlator output
$C_{VL}$	Very Late correlator output

The implementation of the tracking multicorrelator algorithm is shown in Figure 5 and described in Algorithm 2. The FPGA performs the Doppler wipe-off using a NCO carrier generator, and multiplies the incoming signal with several code replicas with configurable spacing between them. The tracking loops use the code replicas to estimate the derivative $\frac{dR_{xd(\tau )}}{d\tau }$ zero-crossing, which is used as a timing error detector [60]. The code replicas are named VE (Very Early), E (Early), P (Prompt), L (Late), and VL (Very Late) [61]. The correlator outputs are integrated and dumped.

**Algorithm 2** Tracking multicorrelator hardware accelerator
1:Initialize the Pilot correlator outputs: $C_{VE_{Pilot}}=0$, $C_{E_{Pilot}}=0$, $C_{P_{Pilot}}=0$, $C_{L_{Pilot}}=0$, and $C_{VL_{Pilot}}=0$2:Initialize the Data correlator output: $P_{Data}=0$3:for n=0 to n=N−14:   Perform Doppler wipe-off: $x\left[n\right]=x_{IN}\left[n\right]\;\cdot \;e^{-j2\pi f_{D}nT_{s}}$5:   For each pilot correlator, determine the following data point of the pilot’s PRN sequence: $c{\left[n\right]}_{Pilot}^{VE},c{\left[n\right]}_{Pilot}^{E},c{\left[n\right]}_{Pilot}^{P},c{\left[n\right]}_{Pilot}^{L},c{\left[n\right]}_{Pilot}^{VL}$6:   For each pilot correlator, determine the following data point of the pilot’s secondary code sequence: $s{\left[n\right]}_{Pilot}^{VE},s{\left[n\right]}_{Pilot}^{E},s{\left[n\right]}_{Pilot}^{P},s{\left[n\right]}_{Pilot}^{L},s{\left[n\right]}_{Pilot}^{VL}$7:   Determine the following data point of the data’s PRN sequence: $c{\left[n\right]}_{Data}^{P}$8:   Determine the following data point of the data’s secondary code sequence: $s{\left[n\right]}_{Data}^{P}$9:   Accumulate the pilot’s correlator outputs:10:      $C_{VE_{Pilot}}=C_{VE_{Pilot}}+x\left[n\right]c{\left[n\right]}_{Pilot}^{VE}s{\left[n\right]}_{Pilot}^{VE}$11:      $C_{E_{Pilot}}=C_{E_{Pilot}}+x\left[n\right]c{\left[n\right]}_{Pilot}^{E}s{\left[n\right]}_{Pilot}^{E}$12:      $C_{P_{Pilot}}=C_{P_{Pilot}}+x\left[n\right]c{\left[n\right]}_{Pilot}^{P}s{\left[n\right]}_{Pilot}^{P}$13:      $C_{L_{Pilot}}=C_{L_{Pilot}}+x\left[n\right]c{\left[n\right]}_{Pilot}^{L}s{\left[n\right]}_{Pilot}^{L}$14:      $C_{VL_{Pilot}}=C_{VL_{Pilot}}+x\left[n\right]c{\left[n\right]}_{Pilot}^{L}s{\left[n\right]}_{Pilot}^{L}$15:Accumulate the data correlator output:16:      $C_{P_{Data}}=C_{P_{Data}}+x\left[n\right]c{\left[n\right]}_{Data}^{P}s{\left[n\right]}_{Data}^{P}$17:end for



The computations of the multicorrelator algorithm are concurrently executed in the FPGA. The coherent integration time can be extended up to the duration of one data symbol without any processor intervention. When using a coherent integration time larger than one data symbol duration, the correlation results have to be further accumulated in software.

The tracking multicorrelators can be configured to use a variable number of code replicas to track the pilot and data components of the GNSS signals. The configuration shown in Table 4 is used. The current implementation of the tracking multicorrelators uses two bits per sample.

**Table 4:** Tracking multicorrelator configuration.

Signal	Configuration
GPS L1 C/A	E, P, L
Galileo E1b/c	VE, E, P, L, VL (Pilot Component)
	P (Data Component)
GPS L5	E, P, L (Pilot Component)
	P (Data Component)
Galileo E5a	E, P, L (Pilot Component)
	P (Data Component)

### 3.3. Software Architecture

**Figure 6:**
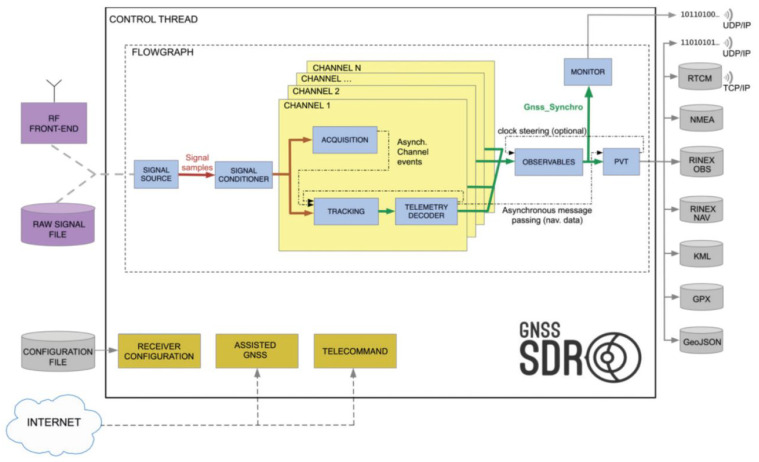
Software architecture of the GNSS-SDR software receiver [11,12].

The software-defined GNSS baseband processing engine is based on GNSS-SDR [11,12]. Figure 6 shows the signal processing flow graph implemented in GNSS-SDR when running on a personal computer. GNSS-SDR implements the baseband signal processing chain, from sample capture up to the computation of the position, velocity, and time (PVT) solution and the generation of GNSS products in standard formats, enabling interoperability and integration with other systems. It consists of a modular design with several processing blocks: signal source, signal conditioner, acquisition, tracking, navigation message decoder, observables, and PVT, enabling easy addition, modification, and replacement of GNSS receiver algorithms. A *Control Thread* reads the GNSS-SDR configuration file and runs in parallel to the flow graph, receiving notifications, and triggering changes in the receiver state. A channel finite state machine (FSM) controls the interaction of the various channel blocks. The source code is based on GNU Radio [62], a widely used free software development framework that provides signal processing functions for implementing software-defined radios. The receiver uses GNU radio’s streaming data flow model and messaging system to pass data and asynchronously communicate events between blocks. The acquisition and tracking blocks are regular GNU radio signal processing blocks, processing the signals generated by the signal sources. The source code is written in C++ and it is released under the GNU General Public Licence (GPL). The software receiver can be built using popular and freely available compilers. As a result, the source code can be freely inspected and modified, and it is portable across multiple architectures, including but not limited to Intel x86-64 and ARM.

**Figure 7:**
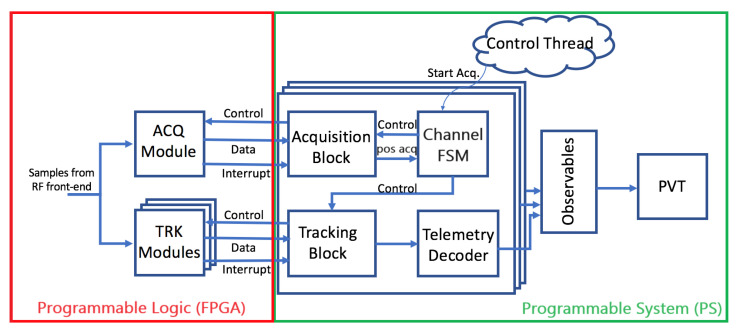
Interface between the FPGA IPs and the GNSS-SDR software receiver.

The use of embedded devices requires the implementation of hardware accelerators in the FPGA for the computationally expensive tasks: the acquisition and the tracking processes [13]. To off-load computational burden from the embedded processor, the FPGA receives and processes the signals coming from the RFFE, replacing the GNSS-SDR’s signal sources, and the flow graph structure is changed as shown in Figure 7. The software signal source blocks are removed, the acquisition block becomes a function that is called when GNSS-SDR proceeds to detect the presence or absence of a satellite signal, and the tracking blocks act as software signal sources, providing the FPGA multicorrelator’s outputs and their In-phase I and Quadrature Q components to the telemetry decoder blocks. The acquisition and the tracking multicorrelator algorithms are performed in the FPGA with no processor intervention, but the tracking phase-locked loops (PLL) and delay-locked loops (DLL) are managed in the software tracking blocks. GNSS-SDR configures and controls the execution of the acquisition and tracking FPGA hardware accelerators by writing and reading data to/from their memory-mapped registers. The acquisition and tracking accelerators launch an interruption to the processing system when they are ready with new data.

## 4. Design Methodology

The prototyping of SoC-FPGA-based systems comprises a software design and a hardware design. The software design involves the programming of GNSS-SDR signal processing blocks, while the hardware design entails the design of the FPGA hardware accelerators. The main existing approaches to design SoC-FPGA–based systems are the following:

- Hardware/software design flow: The system is divided into hardware and software sections that are designed independently, using dedicated tools for each. The designer searches for an optimal partitioning and assignment of tasks between the software running in the embedded processor and the hardware implemented in the FPGA, with the objectives of minimizing power consumption and taking advantage of the FPGA’s massively parallel architecture. Software and hardware development are followed by integration testing, where the FPGA and the software components are combined and tested to confirm that they interact according to their requirements [54].
- Software-oriented, hardware/software co-design flow: the functionality of the whole system is described at a high level of abstraction using software code or block-based design techniques. The tools can then quickly partition hardware and software elements in the SoC-FPGA in different ways according to the designer’s commands, and all the communication interfaces between the FPGA and the software are automatically managed by the tools [54].

The authors use a design methodology based on separate software and hardware design flows. This enables the development of the GNSS-SDR software signal processing blocks using FOSS development tools and a FOSS compiler toolchain, independently of the FPGA development tools. Consequently, the development of novel GNSS receiver algorithms is carried out in two steps, shown in Table 5.

**Table 5:** SoC-FPGA-based GNSS receiver design flow.

Design Step	Explanation
Step 1: Software Design	Implements and validates the GNSS algorithms in software, using GNSS-SDR
Step 2: FPGA Design	Implements the FPGA hardware accelerators. Modifies the GNSS-SDR source code to use the hardware accelerators when the option to use an FPGA is enabled.
Step 3: System Integration	Integrates the software and the FPGA components and tests the whole system in an SoC-FPGA platform

Code reuse is a fundamental aspect of the proposed design methodology. Implementing the first prototype can be a lengthy process involving FPGA development and the configuration and cross-compilation of an embedded GNU/Linux system for the targeted hardware. However, once the first prototype is implemented, adding new features or porting the existing architecture to other SoC-FPGA variants becomes a much quicker process, facilitating research on novel and experimental algorithms. The following subsections explain the software design and the FPGA design steps in more detail.

### 4.1. Software Receiver Design Methodology

**Figure 8:**
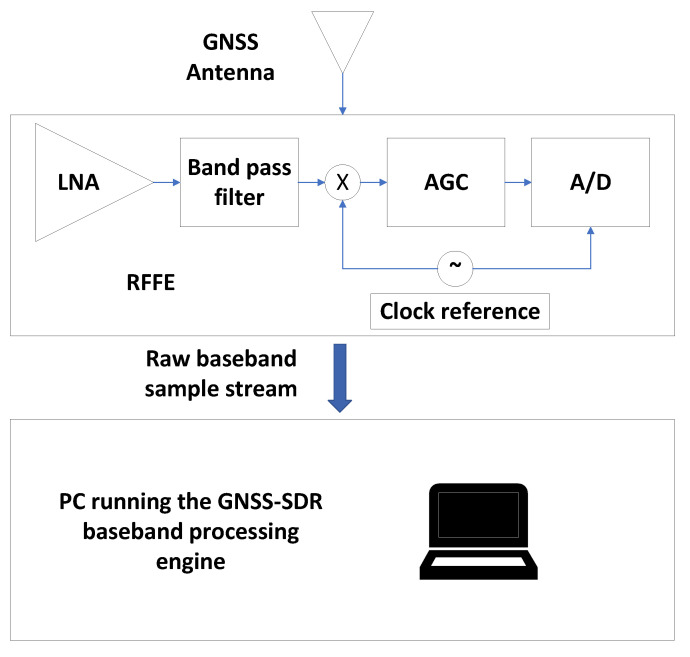
Host-based GNSS-SDR receiver.

Researchers may implement experimental receiver algorithms in the form of GNSS-SDR software blocks [11,12], creating new software blocks or modifying the existing ones. The new software blocks can be easily integrated into GNSS-SDR. By default, GNSS-SDR implements a host-based receiver architecture as shown in Figure 8, where a general-purpose processor, usually a PC, implements the baseband signal processing chain. An external RF tuner featuring a low-noise amplifier (LNA), an automatic gain control (AGC), and an A/D converter can be used to receive live GNSS signals. GNSS-SDR can process GNSS signals in real-time, provided that the processor is fast enough. Thus, researchers can test new signals and algorithms with live signals in real-time using a PC and an analog front-end. If the complexity of the implementation prevents real-time processing, researchers may use GNSS-SDR in post-processing mode using recorded signals.

A GNSS-SDR website is available on the internet [12], containing tutorials, instructions, and examples of how to download, compile, use, and contribute to GNSS-SDR. Researchers are encouraged to contribute to GNSS-SDR and push the newly implemented changes to the GNSS-SDR repository. In this way, novel algorithms are available to the research community, allowing for review, code inspection, and further modification.

When the experimental GNSS receiver algorithms have been validated in software, researchers may require a battery-powered, compact, and portable device to validate the novel GNSS algorithms in the field. An SoC-FPGA-based receiver can be used to meet these requirements.

### 4.2. SoC-FPGA-Based Receiver Design Methodology

**Figure 9:**
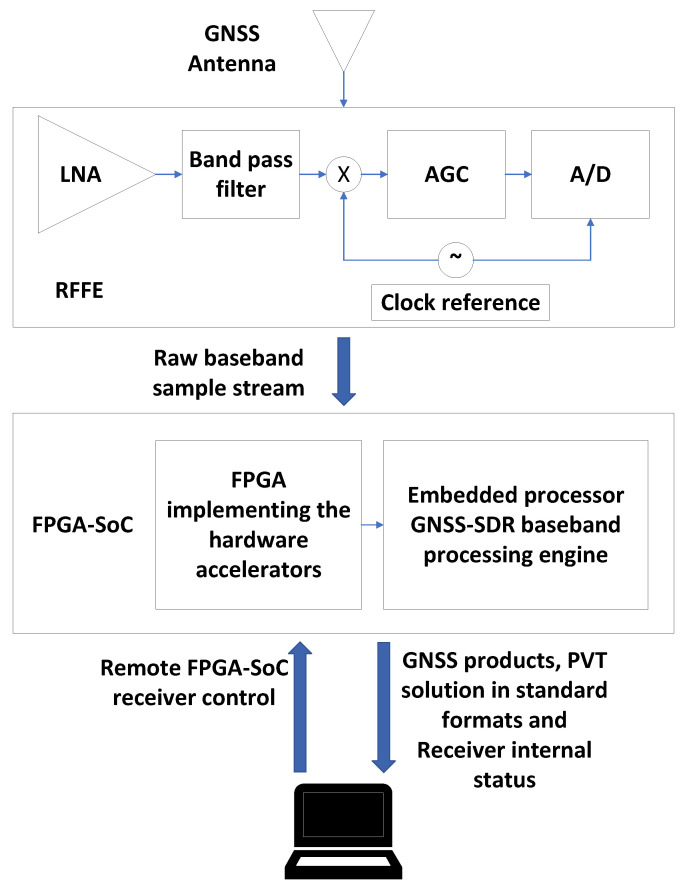
SoC-FPGA GNSS receiver.

An SoC-FPGA-based design implements the receiver shown in Figure 9. In many cases, the RFFE and the SoC-FPGA device can be integrated into the same board, leading to a tightly integrated design. The procedure to design an SoC-FPGA-based receiver involves two steps:

- The first step consists of the implementation of the FPGA design containing the sample conditioning module, the acquisition and tracking modules, and the PS/PL interface shown in Figure 2.
- The second step is the creation of an embedded GNU/Linux system for the SoC embedded processor and a GNU/Linux software development kit (SDK). The SDK is then used to cross-compile GNSS-SDR for the embedded platform.

Many of these design steps can be highly automated using FOSS tools. After the initial implementation is in place, it can be substantially reused many times using different SoC-FPGAs. The subsections below explain the FPGA design process, the creation and configuration of the embedded GNU/Linux system and the SDK, and the GNSS-SDR cross-compilation for the embedded processor in more detail.

#### 4.2.1. FPGA Design Process

The FPGA design process requires the use of specialized FPGA development tools. FPGAs are usually programmed using Hardware Description Languages (HDLs) such as Verilog or Very-High-Speed Integrated Circuit Hardware Description Language (VHDL), at the register transfer level (RTL) of abstraction. The authors implemented proof-of-concept SoC-FPGA GNSS receivers in VHDL using the AMD Zynq-7000 and the Zynq Ultrascale+ families of SoC-FPGAs. Hence, the FPGA design process requires the use of the AMD Vivado FPGA Design Suite [63]. FPGAs can also be programmed using a higher level of abstraction, using software code or a block-based design approach: A process named high-level synthesis (HLS) generates RTL models out of the software specification. Users wishing to implement FPGA hardware accelerators using HLS may use the AMD Vitis Unified Software Platform [64]. The FPGA design tools are not distributed using FOSS licenses. However, the academic and research community, which includes educators, researchers, and students, may be eligible for the resources provided by existing university programs [65].

The FPGA design process is devoted to the implementation of the architecture shown in Figure 3. The design of the FPGA architecture takes place in two steps:

- The first step is the implementation of the acquisition and tracking multicorrelator hardware accelerators. The FPGA hardware accelerators are implemented as separate FPGA projects, and they are packaged in the form of FPGA IP cores. FPGA IPs are pre-designed, reusable blocks of hardware functionality or components that can be integrated into FPGA designs. These IP cores are typically provided by FPGA manufacturers, third-party vendors, or open-source communities to simplify the development of complex FPGA-based systems
- The second step is the creation of an FPGA project implementing the sample conditioning, the PS/PL interface blocks, and the sample buffers shown in Figure 3. The designer imports the required acquisition and tracking multicorrelator FPGA IP cores into the FPGA project and connects them to the PS/PL interface and to the sample buffers if necessary.

Researchers need to determine whether novel GNSS receiver algorithms need to be implemented in the FPGA in the form of FPGA hardware accelerators. This usually depends on two factors: the algorithm’s computational load and whether the algorithm directly processes the samples coming from the analog front-end. Algorithms requiring a high computational load or directly processing the samples coming from the analog front-end cannot run in the embedded processor when the embedded processor is running GNSS-SDR in real time. As opposed to this, signal processing blocks on the right side of the flow graph shown in Figure 6, between the tracking multicorrelators and the computation of the PVT solution, can usually be implemented in software. If the researcher’s experimental algorithms require the implementation of an FPGA hardware accelerator, then the algorithms can be implemented in a separate FPGA project and packaged in the form of an FPGA IP core, in the same way as the acquisition and tracking multicorrelators.

Some manufacturers provide FPGA example designs implementing RFFE interfaces and DMA blocks. For instance, Analog Devices, provides FPGA example designs, libraries, and projects under FOSS licenses for various FPGA architectures [66]. These designs can be reused, reducing time and effort.

#### 4.2.2. Configuring and Building the Embedded GNU/Linux System and Cross-Compiling GNSS-SDR for the Embedded Platform

The authors use the Petalinux tools [67] to configure and build an embedded GNU/Linux system and an SDK for the target SoC-FPGA platform. The Petalinux tools are based on the Yocto project [68]. The Yocto project is an open-source building framework for the creation of customized embedded GNU/Linux systems, providing tools, processes, templates, and methods to rapidly create and deploy embedded platforms. The Petalinux tools are a set of high-level commands that are built on top of the Yocto Linux distribution enabling the customization, build, and deployment of embedded Linux systems for various SoC-FPGA variants, including the Zynq-7000 and the Zynq Ultrascale+ families. The process to configure and build an embedded GNU/Linux system, build an SDK, and cross-compile GNSS-SDR for the embedded platform involves the steps below:

- The first step is to create a Petalinux project for the target SoC-FPGA platform
- The second step is to import the compiled FPGA design into the Petalinux project.
- The third step is to configure the embedded GNU/Linux system. The designers shall add the software libraries that are essential for the execution of GNSS-SDR, and any other software libraries that may be considered. This step is usually performed using the Petalinux tools. The designers may also configure the device tree, describing the various hardware accelerators that are accessible from the GNU/Linux system. The device tree is a file containing information about the board and its hardware, including the FPGA hardware accelerators.
- The fourth step is to cross-compile the GNU/Linux system for the embedded processor. The cross-compiled system contains the boot files, the OS kernel and file system, the FPGA bitstream describing the FPGA logic, and a device tree blob, which is a compiled version of the device tree.
- The fifth step is to create a software development kit (SDK). This can be done using Petalinux commands.
- The last step is to use the SDK to cross-compile GNSS-SDR for the embedded processor, using the appropriate flags telling the system to offload the computationally expensive signal processing functions to the FPGA.

Instructions on how to create custom SDKs and how to cross-compile GNSS-SDR using the custom SDKs are available in [12]. After building GNSS-SDR and the embedded GNU/Linux system, the designers need to copy the cross-compiled system into a non-volatile memory such as an SD card. The SoC-FPGA receiver uses the non-volatile memory for the boot process. Finally, once the device is booted, users can remotely log into the system and run GNSS-SDR.

## 5. Proof of Concept Demonstrators

The authors designed three proof-of-concept demonstrators to test the potential of the proposed architecture. A spaceborne GNSS receiver and a GNSS rebroadcaster were reported in [15] and [16], respectively, extending the capabilities of the architecture to the real-time generation and regeneration of GNSS signals. The newly introduced general-purpose GNSS receiver acts as a platform for assessing performance and conducting future experiments. Table 6 shows the components that were used for the implementation of the prototypes.

**Table 6:** Proof-of-concept demonstrators.

Device	Board	SoC-FPGA Platform	RFFE	Size
Spaceborne GNSS receiver [15]	ADRV9361-Z7035 Evaluation Board [69]	Zynq 7000 XC7Z035-L2 FBG676I [56]	AD9361 RF Agile Transceiver [70] (integrated in the evaluation board)	100 mm × 62 mm
GNSS Rebroadcaster [16]	ADRV9361-Z7035 Evaluation Board [69]	Zynq 7000 XC7Z035-L2 FBG676I [56]	AD9361 RF Agile Transceiver [70] (integrated in the evaluation board)	100 mm × 62 mm
General Purpose GNSS Receiver	Zynq UltraScale+ MPSoC ZCU102 Development Board [71]	Zynq ULtrascale+ XC7ZU9EG-2FFVB1156E [55]	AD-FMCOMMS5-EBZ [72]	23.749 cm × 24.384 cm (ZCU102 Board), 14 cm × 9 cm (RFFE)

Both the spaceborne GNSS receiver and the GNSS rebroadcaster were implemented using the Analog Devices ADRV9361-Z7035 evaluation board [69], which integrates an Analog Devices’ AD9361 RF Agile Transceiver [70] and AMD’s Zynq 7000 XC7Z035-L2-FBG676I all programmable SoC [56] housing a dual-core ARM Cortex-A9 processor running at 800 MHz and a Kintex-7 FPGA with 275 k logic cells and 900 DSP48 slices. The on-board clock shipped with the ADRV9361-Z7035 evaluation board was replaced with a pin-compatible high-precision temperature-compensated crystal oscillator by TXC Crystal, model TXC 7Q series for GPS-specific applications, clocked at 40 MHz with a frequency stability of 2 ppm [15]. The ADRV9361-Z7035 is a small board, integrating the RFFE and the SoC-FPGA in a 100 mm × 62 mm platform. The ADRV9361-Z7035 was attached to an ADRV1CRR-BOB breakout board to provide easy access to user I/O, Ethernet, USB, JTAG, and serial connections.

The newly unveiled general-purpose GNSS receiver is implemented using AMD’s Zynq UltraScale+ MPSoC ZCU102 Evaluation Board [71], featuring an Zynq ULtrascale+ XC7ZU9EG-2FFVB1156E MPSoC [55], containing a quad-core ARM Cortex-A53 processor running at 1200 MHz and an FPGA with 600 k logic cells and 2520 DSP slices. The RFFE is implemented using an Analog Devices AD-FMCOMMS5-EBZ analog front-end featuring two Analog Devices’ AD9361 RF Agile Transceivers [72]. The AD-FMCOMMS5-EBZ board is shipped with a RXO3225M IC crystal by Rakon, clocked at 40 MHz, with a stability of 25 ppm, which is not enough for GNSS signal processing. Thus, using an external oscillator is mandatory. The authors use an external Abracon AST3TQ-50 reference oscillator [73], with a frequency stability of 50 ppb over −40 °C to +85 °C operating temperature range, and it is well suited for GNSS applications. The ZCU102 is a large board, containing a large variety of peripheral interfaces, measuring 23.749 cm × 24.384 cm, not taking into account the RFFE. The purpose of this device is to serve as a testbed to implement new features and enhance the proposed architecture.

A summary of the most relevant features of the proof-of-concept implementations and the achieved results are provided in the subsections below.

### 5.1. Spaceborne GNSS Receiver

**Figure 10:**
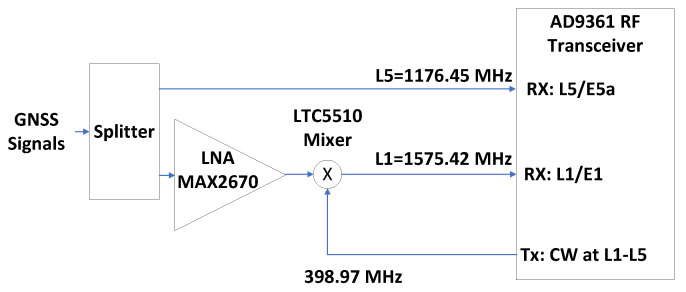
Making the AD9361 a dual frequency receiver [15].

The spaceborne GNSS receiver prototype [15] is a dual-band (centered at 1176.5 and 1575.42 MHz) receiver with the ability to process GPS L1 C/A, Galileo E1b/c, GPS L5, and Galileo E5a signals. The RFFE is implemented using an Analog Devices AD9361 RF Agile Transceiver [70], which does not allow tuning each channel to a different frequency band. To enable dual-band reception, one of the AD9361’s transmission channels is set to generate a continuous wave (CW) signal tuned at the difference between L1/E1 and L5/E5a center frequencies (that is, 398.97 MHz), and this signal is used to shift the L1/E1 band down to the L5/E5a center frequency. Hence, both AD9361 transmission channels can work at the same center frequency (that is, $f_{L5}=1176.45$ MHz), while the whole system is still acting as a dual-band RF front-end. This is shown in Figure 10.

This device demonstrates the implementation of a multi-frequency, multi-constellation small form factor (SFF) GNSS receiver measuring 100 mm × 62 mm, capable of processing GNSS signals using high dynamic low-Earth-orbit scenarios. The SoC-FPGA’s power usage is projected to be 6.5 W, as per the FPGA design tools’ assessment.

### 5.2. GNSS Rebroadcaster

The rebroadcaster [16] is a GNSS signal regenerator that can generate, or receive and regenerate GNSS signals both in post-processing mode and in real-time with very low latency. The rebroadcaster can regenerate the received satellite signals in real-time while simultaneously rebroadcasting a PVT solution that differs from the PVT fixes obtained by the SW receiver. This device can be used to test the addition of new features in GNSS signals, and to characterize the performance of new and existing spoofing detection algorithms. The low latency facilitates maintaining the consistency with other sensors, e.g., an inertial measurement unit, where available.

The rebroadcaster comprises a GNSS receiver and a GNSS transmitter in a single SoC-FPGA. The FPGA implements hardware accelerators both for the receiver and the transmitter parts of the rebroadcaster. The receiver processes GNSS signals, obtains the satellite’s ephemeris data, and computes PVT. The transmitter is implemented in software, running as a separate application in the embedded processor, as well as in hardware. The software transmitter implements an orbit engine and an observables engine. The orbit engine uses the satellite’s ephemeris data to compute the position of the satellites that are visible from the retransmitted PVT solution. This is followed by the observables engine, which computes the GNSS observables relative to the transmitted PVT solution. Finally, the software transmitter computes the shape of the transmitted signals (code phases, carrier phases, phase steps, telemetry data, etc.) and sends a continuous command stream to the FPGA with all this data. The FPGA implements various signal generator hardware accelerators, where each signal generator is assigned to a satellite. The FPGA generates the digitized transmitted signals according to the commands received from the software receiver.

**Figure 11:**
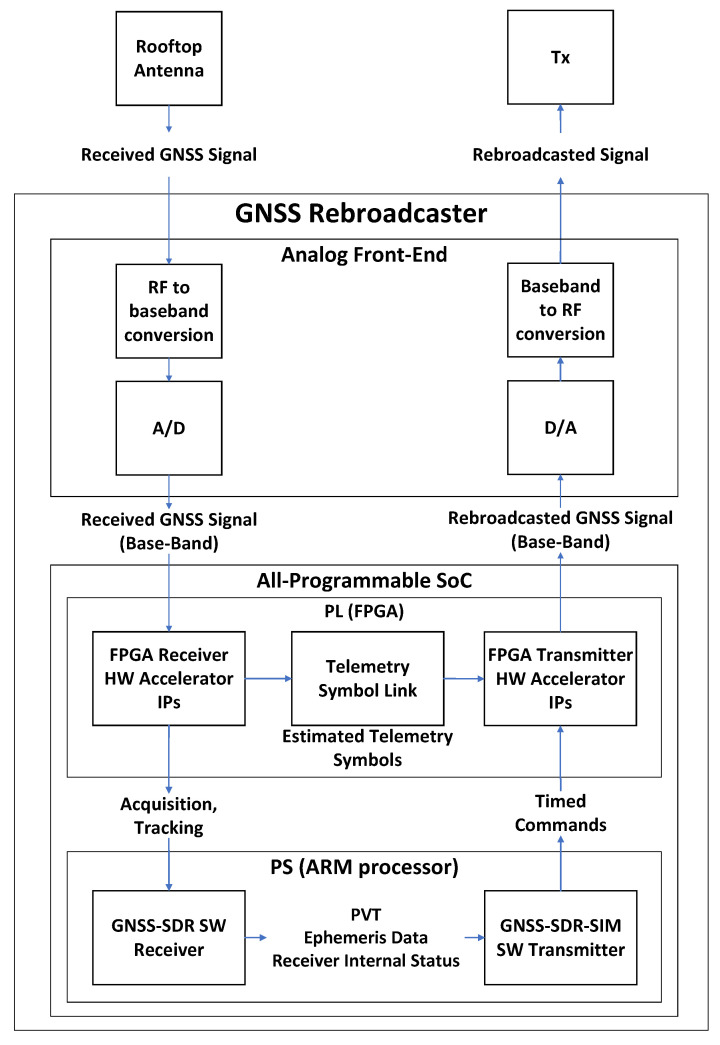
GNSS signal regenerator block diagram [16].

The GNSS rebroadcaster implements a special feature in the FPGA named telemetry symbol link. The telemetry symbol link forwards the telemetry data estimated in the receiver tracking multicorrelators to the signal generators in the FPGA without any processor intervention. Figure 11 shows the telemetry symbol link acting as a fast path from the FPGA tracking multicorrelators to the FPGA transmitter channels without having to go through the slower path in the processing system. When the telemetry symbol link is enabled, the telemetry data is rebroadcasted with a latency below 30 ms.

The rebroadcaster is tested using GPS L1 C/A and Galileo E1b/c signals. The telemetry symbol link can only be used with the Galileo E1b/c signals.

The real-time signal generation and regeneration of GNSS signals was demonstrated using GPS L1 C/A and Galileo E1b/c signals. The telemetry symbol link was demonstrated using Galileo E1b/c signals [16]. The SoC-FPGA’s power usage is estimated to be 5.38 W, as per the FPGA design tools’ assessment.

### 5.3. General Purpose GNSS Receiver

The general-purpose GNSS receiver introduced in this paper implements a multi-band, multi-frequency device capable of processing GPS L1 C/A, Galileo E1b/c, GPS L5 and Galileo E5a signals in real-time and in post-processing mode. The receiver implements 48 multicorrelator hardware accelerators, potentially tracking up to 12 GPS L1 C/A, 12 Galileo E1b/c, 12 GPS L5, and 12 Galileo E5a signals when running in post-processing mode. When running in real-time mode, the maximum number of signals that the receiver can track is currently limited by the computing power of the embedded processor.

**Figure 12:**
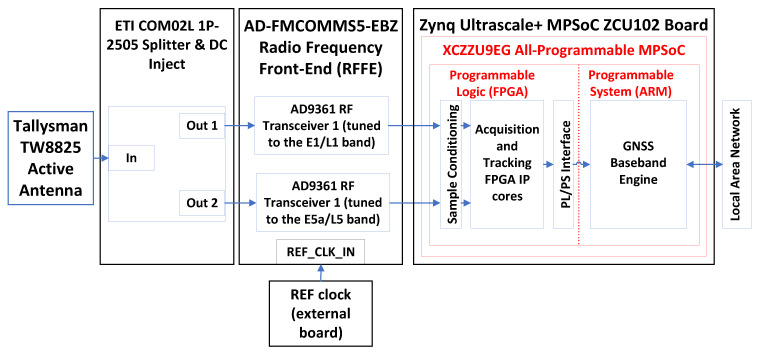
General purpose GNSS receiver block diagram.

A block diagram of the proposed receiver is shown in Figure 12. A Tallysman TW8825 active antenna [74] receives the GNSS signals from the sky. A splitter injects DC power to the active antenna and splits the signal in two. An AD-FMCOMMS5-EBZ RFFE [72] featuring two Analog Devices AD9361 RF Agile Transceivers [70] receives the signals coming from the splitter. One RF transceiver is tuned to the L1/E1 frequency band, and the other transceiver is tuned to the L5/E5a frequency band.

The ZCU102 development board was chosen because it features many peripherals and interfaces, enabling development and experimentation for a wide range of GNSS applications.

## 6. Results

The experimental results shown in this section were obtained using the newly introduced general-purpose GNSS receiver, implemented using the [71] Zynq Ultrascale+ MPSoC ZCU102 Development board. The authors assess the receiver sensitivity, the number of channels that the receiver can simultaneously track when working in real-time, multi-frequency, and multi-constellation modes, the quality of the observables, the precision and accuracy of the navigation solutions, and the ability of the receiver to process GNSS signals in LEO scenarios. Appendix A provides the receiver acquisition, tracking, and PVT configuration used to obtain the experimental results when using static scenarios. Appendix B shows the receiver configuration used to measure the accuracy of the navigation solutions when using an LEO scenario.

### 6.1. Receiver Sensitivity

Receiver sensitivity is a measure of the minimum signal strength that a receiver can detect. The authors measured the acquisition and tracking sensitivity using a signal generator. The acquisition and tracking sensitivity measurements are explained in the subsections below. The receiver was configured according to Appendix A.

#### 6.1.1. Acquisition Sensitivity

The authors measured the acquisition sensitivity as the minimum $C/N_{0}$ that a GNSS signal needs to have for the receiver to lock onto it for the first time. The acquisition sensitivity was measured as follows: the receiver was connected to a signal generator. The signal generator was playing recorded GNSS signals. The receiver was running in real time. The authors decreased the signal power and recorded the lowest $C/N_{0}$ for which the receiver could successfully acquire and track the detected signals. The receiver was set in cold start mode before performing each measurement. The $C/N_{0}$ reported by the receiver was recorded when the receiver went from acquisition to tracking mode. The results are shown in Table 7.

**Table 7:** Acquisition sensitivity.

GNSS System	Acquisition Sensitivity (dB-Hz)
GPS L1 C/A	37
Galileo E1b/c	39
GPS L5	38
Galileo E5a	38

To complement these measurements, the authors moved the receiver outdoors using the Tallysman TW8825 active antenna and verified that the receiver could successfully acquire most of the GNSS satellites that were visible and in the line of sight. However, the current acquisition sensitivity is a bit too high, and the receiver may have difficulties detecting satellites that are at low elevation angles. The reason for this is that the current version of the FPGA acquisition hardware accelerator integrates over one PRN code period and does not perform any type of extended coherent and/or noncoherent integration.

#### 6.1.2. Tracking Sensitivity

The authors measured the tracking sensitivity as the minimum $C/N_{0}$ that a GNSS signal needs to have for the receiver to track the signal. The tracking sensitivity was measured as follows: the receiver was connected to a signal generator. The signal generator was playing recorded GNSS signals. The receiver was running in real time. When the receiver was in tracking mode, the authors decreased the signal power and recorded the lowest $C/N_{0}$ for which the receiver could successfully track the detected signals. The $C/N_{0}$ reported by the receiver was recorded. The receiver was configured according to Appendix A. The results are shown in Table 8.

**Table 8:** Tracking sensitivity.

GNSS System	Acquisition Sensitivity (dB-Hz)
GPS L1 C/A	26
Galileo E1b/c	28
GPS L5	29
Galileo E5a	29

The current tracking sensitivity is sufficient for the receiver to track the visible GNSS satellites that are visible and in the line of sight at all elevation angles.

### 6.2. Multi-Frequency and Multi-Constellation Operating Modes

The authors assessed the receiver’s capability to process various satellite signals in real time, including GPS L1 C/A, Galileo E1b/c, GPS L5, and Galileo E5a. The receiver operated in selectable modes, accommodating single or dual frequencies, as well as single or dual constellations, with a potential of up to 12 channels per signal. To augment satellite visibility, the receiver was connected to the GESTALT testbed rooftop GNSS antenna facility [7]. The receiver was allowed to simultaneously track the same signals across multiple channels, ensuring that each channel was allocated to a satellite signal. The testing duration lasted for an hour. The receiver was configured according to Appendix A. The receiver was tested to acquire and track the satellite signals, demodulate the navigation messages, and compute PVT in real time, using the signal combinations shown in Table 9.

**Table 9:** GNSS signal combinations tested.

GNSS Signals	Total Number of Signals
12 GPS L1 C/A	12
12 Galileo E1b/c	12
12 GPS L5	12
12 Galileo E5a	12
12 GPS L1 C/A + 12 Galileo E1b/c	24
12 GPS L5 + 12 Galileo E5a	24
12 GPS L1 C/A + 12 GPS L5	24
12 Galileo E1b/c + 12 Galileo E5a	24
10 GPS L1 C/A + 10 Galileo E1b/c + 10 GPS L5 + 10 Galileo E5a	40

The number of signals the receiver could simultaneously track was restricted by the computational capabilities of the embedded processor. While the FPGA handled acquisition and tracking multi-correlations, the embedded processor managed the tracking loops, with the FPGA generating interrupt requests for this purpose. The rate of interrupt requests was directly related to the number of tracked channels, and an excessive number of interrupts could potentially overwhelm the embedded processor. It would be possible to enhance receiver performance by making adjustments to the multicorrelator tracking hardware accelerators in the FPGA. Extending the coherent integration time within the FPGA beyond that of a single data symbol would lead to a reduction in the frequency of interrupt requests, especially when tracking Galileo E1b/c signals, known for their brief data symbol duration of 4 ms. Implementing this strategy would consequently enable the receiver to track more GNSS signals simultaneously.

### 6.3. Observables Quality

The authors measured the root mean square error (RMSE) of the receiver carrier phase estimation and the RMSE of the receiver code phase estimation and compared the measurements against the theoretical variance of the carrier phase and code phase estimates.

A software GNSS simulator created by the authors and available online under the GPL v3 license [75] was used for this test. The RMSE of the receiver carrier phase estimation and the RMSE of the receiver code phase estimation were measured as follows: The authors produced synthetic GPS L1 C/A signals at various signal-to-noise ratios (SNRs). The satellite signals were generated in pairs, using identical ephemeris information and satellite locations but different PRN numbers for each pair of satellites. The synthetic signals were generated in baseband and stored in files (no RF was used). The GNSS receiver processed these files and produced the basic observable measurements (pseudorange, carrier phase, and Doppler shift). The observables were stored in RINEX files. Then, the between-satellites single difference was computed for each satellite pair. In this way, the code delay errors and the code phase estimation errors were obtained. The RMSE of the carrier phase estimations and the RMSE of the code phase estimations were computed out of the estimation errors. Finally, the RMSE measurements were compared against the theoretical variance of the carrier phase and code phase estimates.

The authors used Equations (1) and (2) to compute the theoretical variance of the carrier phase delay estimates, and the theoretical variance of the code phase delay estimates, respectively. Equation (1) can be used to compute the thermal noise carrier tracking jitter when using an arctangent PLL discriminator [60], and Equation (2) can be used to compute the thermal noise code tracking jitter when using a noncoherent early-late power DLL discriminator [60]. Equation (2) is applicable for direct sequence spread spectrum (DSSS) signals generated using BPSK (binary phase-shift keying) signaling with rectangular chips such as the C/A code [60]. In all cases, the thermal noise is treated as the only source of error.

The parameters in Equations (1) and (2) are the following: $\lambda_{L1}$ is the GPS L1 carrier wavelength in m, *c* is the speed of light in vacuum, $C/N_{0}$ is the carrier to noise density ratio, $T_{int}$ is the pre-detection integration time in s, δ is the early-to-late correlator spacing in chips, $BW_{PLL}$ is the PLL bandwidth in Hz, $BW_{DLL}$ is the DLL bandwidth in Hz, $B_{fe}$ is the front-end bandwidth in Hz (which in our case it is the same as the sampling frequency), and $T_{c}$ is the chip period in seconds = $1/R_{c}$, where $R_{c}$ is the chipping rate.

(1) $$\sigma_{PLL}=\frac{\lambda_{L1}}{2\pi }\sqrt{\frac{BW_{PLL}}{C/N_{0}}\left(1+\frac{1}{2T_{int}C/N_{0}}\right)}\;\mathrm{[m]}$$

(2) $$\begin{array}{c} \sigma_{DLL}=\left\{\begin{array}{cc} cT_{c}\sqrt{\frac{BW_{DLL}}{2C/N_{0}}\delta \left(1+\frac{2}{T_{int}\left(C/N_{0}\right)\left(2-\delta \right)}\right)} & \mathrm{if}\;\delta \geq \frac{\pi R_{c}}{B_{fe}}\\ cT_{c}\sqrt{\frac{BW_{DLL}}{2C/N_{0}}\left(\frac{1}{B_{fe}T_{c}}+\frac{B_{fe}T_{c}}{\pi -1}{\left(\delta -\frac{1}{B_{fe}T_{c}}\right)}^{2}\right)\left(1+\frac{2}{T_{int}\left(C/N_{0}\right)\left(2-\delta \right)}\right)} & \mathrm{if}\;\frac{R_{c}}{B_{fe}}<\delta <\frac{\pi R_{c}}{B_{fe}}\\ cT_{c}\sqrt{\frac{BW_{DLL}}{2C/N_{0}}\left(\frac{1}{B_{fe}T_{c}}\right)\left(1+\frac{1}{T_{int}C/N_{0}}\right)} & \mathrm{if}\;\delta \leq \frac{R_{c}}{B_{fe}} \end{array}\right. \end{array}\;\mathrm{[m]}$$

The theoretical variance of the code phase estimation was also computed using the simplified calculation shown in Equation (3) [76]. This approximation is applicable when $BW_{DLL}T_{i}\ll 1$. The GNSS receiver was configured using $DLL_{BW}=5$ Hz and $T_{i}$ = 20 ms. Therefore, $DLL_{BW}T_{i}\ll 1$ and Equation (3) can be used.

(3) $$\sigma_{DLL}=cT_{c}\sqrt{\frac{BW_{DLL}}{2C/N_{0}}\delta \left(1+\frac{1}{T_{i}C/N_{0}}\right)}\;\mathrm{[m]}$$

The receiver was configured according to Appendix A, but only GPS L1 C/A channels were enabled. In this test, the receiver was configured with 16 GPS L1 C/A channels to facilitate between-satellite single-difference measurements, utilizing some of the tracking multicorrelator hardware accelerators typically assigned to Galileo E1b/c signals for GPS. The sampling frequency was set to 12.5 Msps, which is sufficient to process GPS L1 C/A signals.

The authors repeated the RMSE measurements both using the FPGA-SoC receiver and using GNSS-SDR running on a PC in a pure software mode (no hardware accelerators involved). The reason is that the FPGA hardware accelerators perform calculations using fixed-point arithmetic, whereas the computations on PCs and embedded processors are carried out using floating-point arithmetic. The employment of fixed point arithmetic can lead to reduced performance during implementation, owing to signal and variable quantization. The results obtained using the FPGA-SoC receiver and using GNSS-SDR in pure software mode were compared to estimate possible implementation losses caused by the fixed point arithmetic in the FPGA. The results are explained in the subsections below.

#### 6.3.1. RMSE of the Carrier Phase

**Figure 13:**
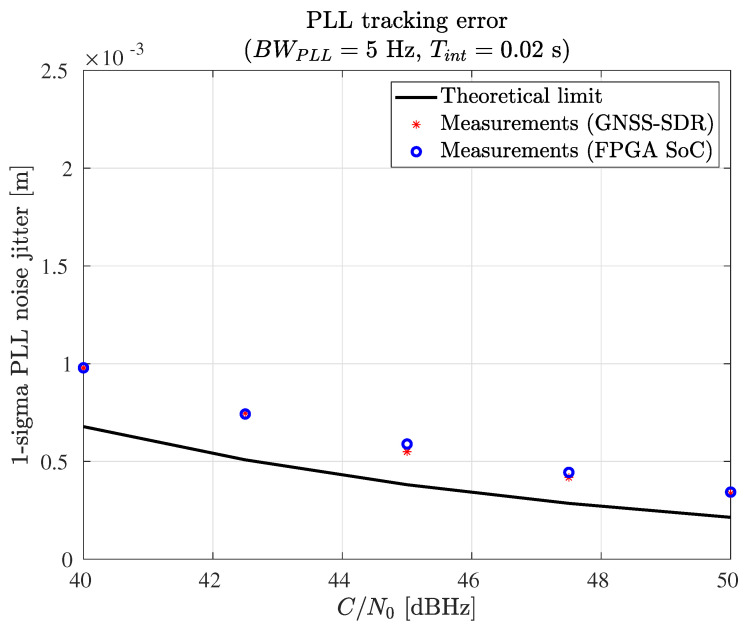
Receiver carrier phase RMSE (1-sigma) when using the FPGA-SoC (stars) and when using GNSS-SDR in software mode (circles).

Figure 13 illustrates the RMSE measured for the carrier phase, alongside the theoretical variance of the carrier phase estimates. The RMSE measured using the FPGA-SoC receiver is shown in circles. The RMSE measured when using GNSS-SDR running in software mode (no hardware accelerators involved) is shown in stars.

Figure 13 indicates an implementation loss between 0.3 dB and 0.5 dB when employing the FPGA hardware accelerators with respect to GNSS-SDR running in pure software mode. This phenomenon may be attributed to the fixed point arithmetic within the FPGA or the action of the FPGA’s dynamic bit selector. The tracking hardware accelerators employ two-bit signal quantization. The FPGA’s dynamic bit selector dynamically adjusts the quantization of the recorded GNSS signals to match the dynamic range of the acquisition and tracking hardware multicorrelators. The aim is to achieve the most efficient quantization possible. The theoretical variance of the carrier phase estimates shown in Figure 13 only considers the presence of thermal noise. However, the difference between the measured values and the theoretical variance suggests that enhancements could be made to refine the estimator’s variance.

#### 6.3.2. RMSE of the Code Phase

**Figure 14:**
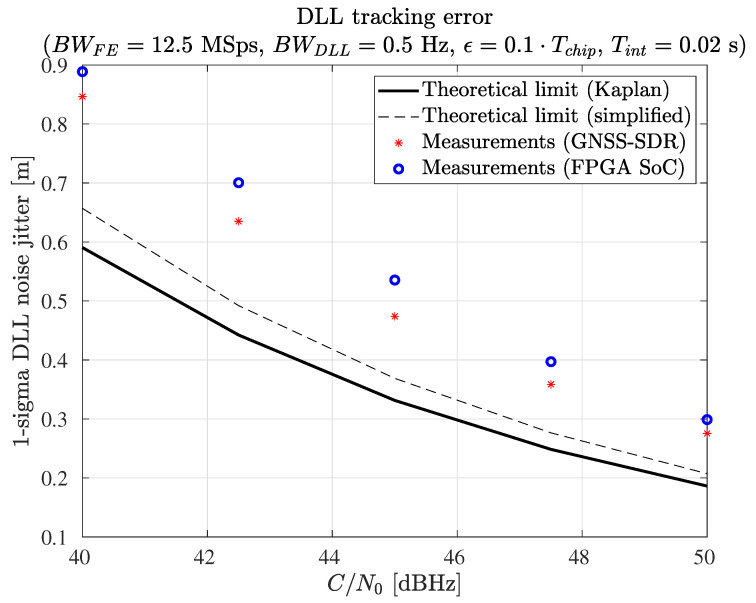
Receiver code phase RMSE (1-sigma) when using the FPGA-SoC (stars) and when using GNSS-SDR in software mode (circles).

Figure 14 illustrates the RMSE measured for the code phase, alongside the theoretical variance of the code phase estimates. The RMSE measured using the FPGA-SoC receiver is shown in circles. The RMSE measured when using GNSS-SDR running in software mode (no hardware accelerators involved) is shown in stars. The theoretical variance of the code phase estimates was computed using Equation (2). The simplified form, designated as Equation (3), was also employed for comparative analysis.

Figure 14 suggests a reduction in performance by approximately 0.5 dB and 1 dB. when utilizing the FPGA hardware accelerators with respect to GNSS-SDR running in pure software mode. The loss may be explained by the quantization of the signals and variables that take place in the FPGA hardware accelerators. The theoretical variance of the code phase estimation shown in Figure 14 only considers the presence of thermal noise. However, the difference between the measured values and the theoretical variance suggests that enhancements could be made to refine the estimator’s variance.

### 6.4. Precision of the Navigation Solutions

The authors performed an assessment of the navigation solutions provided by the receiver using standard positioning precision measurements with deviation maps and their corresponding static confidence regions. Precision refers to how close a solution is to the mean of all the obtained solutions (thus related to the repeatability, or the spread of the measure). Two of the most commonly used confidence measurements for positioning are the distance root mean square (DRMS) and the circular error probability (CEP), for 2D positioning, and the spherical accuracy standard (SAS), the mean radial spherical error (MRSE), and the spherical error probable (SEP), for 3D positioning. Table 10 provides the formulas for standard 2D position confidence regions, while Table 11 contains the formulas for typical 3D position confidence regions [77,78].

**Table 10:** Most common 2D precision measures.

Measure	Formula	Confidence Region Probability
2D 2DRMS	$2\sqrt{\sigma_{E}^{2}+\sigma_{N}^{2}}$	95%
2D DRMS	$\sqrt{\sigma_{E}^{2}+\sigma_{N}^{2}}$	65%
2D CEP	$0.62\sigma_{N}+0.56\sigma_{E}$, if $\frac{\sigma_{N}}{\sigma_{E}}>0.3$	50%

**Table 11:** Most common 3D precision measures.

Measure	Formula	Confidence Region Probability
3D 99% SAS	$1.122(\sigma_{E}+\sigma_{N}\sigma_{U})$	99%
3D 90% SAS	$0.833(\sigma_{E}+\sigma_{N}\sigma_{U})$	90%
3D MRSE	$\sqrt{\sigma_{E}^{2}+\sigma_{N}^{2}+\sigma_{U}^{2}}$	61%
3D SEP	$0.51(\sigma_{E}+\sigma_{N}\sigma_{U})$	50%

The latitude, longitude, and height coordinates obtained by the receiver were converted to a local east–north–up (ENU) coordinate system. The ENU system was anchored to a selected reference point in proximity to the receiver’s antenna, employing a World Geodetic System (WGS)-84 reference ellipsoid. When measuring precision, standard deviations are computed as shown in (4), where E[n] are the east (E) coordinates provided by the receiver, $\bar{E}$ is the measured mean value of the east coordinates, and *L* is the number of available position fixes. The standard deviation for the north (N) and up (U) coordinates can be computed in the same way.

(4) $$\sigma_{E}^{\left(precision\right)}=\sqrt{\frac{1}{L-1}\sum_{l=1}^{L}{\left(E\left[n\right]-\bar{E}\right)}^{2}}\;\mathrm{[m]}$$

The measurements were performed using the GESTALT testbed rooftop antenna [7]. The receiver was configured to process the incoming signals in real time in multi-frequency and multi-constellation mode, using GPS L1 C/A, Galileo E1b+c, GPS L5, and Galileo E5a signals. The receiver was configured according to Appendix A. The sampling frequency was set to 16 Msps. The receiver was also configuread to dump the PVT solutions onto a file. The PVT block was configured to use single-point positioning mode. During the first 15 seconds, the navigation solutions were excluded from consideration to allow the receiver to reach a steady state. The authors performed two tests, each one lasting for 10 minutes. The 2D precision results are shown in Table 12, and the 3D precision results are shown in Table 13. The 2D precision measurements are more precise than the 3D precision measurements. This is expected, as GNSS is more accurate in the horizontal plane than in the vertical plane. This discrepancy arises from the angle between the line of sight to different GPS satellites and the ground. More accurate results may be obtained by conducting extended measurements.

**Table 12:** 2D precision results.

Measure	Results (Test 1) [m]	Results (Test 2) [m]	Confidence Region Probability
2D 2DRMS	6.9	4.9	95%
2D DRMS	3.4	2.4	65%
2D CEP	2.8	2.0	50%

**Table 13:** 3D precision results.

Measure	Results (Test 1) [m]	Results (Test 2) [m]	Confidence Region Probability
3D 99% SAS	9.6	8.6	99%
3D 90% SAS	7.1	6.4	90%
3D MRSE	5.1	4.9	61%
3D SEP	4.3	3.9	50%

### 6.5. Accuracy of the Navigation Solutions Using an LEO Scenario

A signal generator was used to produce a synthetic LEO scenario and a reference motion file. The reference motion file contained the reference positions and timings of the LEO scenario. The receiver was configured to work in high dynamics scenarios. Appendix B shows the receiver configuration for the LEO scenario. The sampling frequency was set to 12.5 MSps. The receiver processed the LEO synthetic scenario in post-processing mode, using a recorded file. The duration of the recorded file was five minutes. The PVT solutions produced by the receiver were recorded in files. The PVT solutions obtained by the receiver were compared against the solutions stored in the reference motion file. The RMSE of the PVT solutions was computed using the Earth-centered, Earth-fixed (ECEF) coordinate system, along with the mean error and the standard deviation.

The results are shown in Table 14. The navigation solutions are fairly accurate. The 3D position RMSE is 1.2 m and the 3D Velocity RMSE is within 0.2 m/s. The RFFE was not used for this test, since the receiver was processing recorded signals.

**Table 14:** PVT accuracy using an LEO scenario.

Measure	Result
3D Position RMSE	1.2 m
3D Position mean error	1.1 m
3D Position standard deviation	0.5 m
3D Velocity RMSE	0.2 m/s
3D Velocity mean error	0.2 m/s
3D Velocity standard deviation	0.1 m/s

### 6.6. Power Consumption

The power consumption of the XCZU9EG-FFVB1156-2-e SoC-FPGA implementing the GNSS receiver is estimated to be 8 W using the FPGA design tools. This estimation assumes an average processor load of 75%, which applies when the receiver manages a significant quantity of GNSS signals. The estimated power usage slightly exceeds that of the spaceborne GNSS receiver [15] and the rebroadcaster [16] proof-of-concept implementations. This result aligns with our intended goals, given that the general-purpose GNSS receiver discussed in this paper utilizes additional FPGA resources and is built with a larger FPGA and a more robust embedded processor [55].

## 7. Conclusions

This work presented an SoC-FPGA architecture and a design methodology for prototyping experimental GNSS receivers, addressing the flexibility limitations of commercial devices. The suggested approach combines the functionalities of both an FPGA and an embedded processor, enabling the implementation of small, portable GNSS receivers. The embedded processor operates GNSS-SDR, a well-known open-source software GNSS receiver, and integrates an FPGA to offload the most computationally demanding tasks from the primary processor.

The authors employ a design approach founded on a hardware/software design flow, allowing separate progression of FPGA and software development. This approach facilitates software implementation through an extensive FOSS toolchain, decoupled from the FPGA development tools. In the proposed methodology, novel receiver algorithms are implemented in two steps: initially, the algorithms are implemented and tested in software, followed by the implementation of hardware accelerators in the FPGA. Inspection of any internal aspect of the receiver relies on the availability of the software version of the algorithms within GNSS-SDR, alongside the documentation offered by the FPGA IP core developers. While the suggested design is compatible with a large number of models of SoC-FPGAs from a single manufacturer, it relies on FPGA IP cores exclusive to that vendor. Hence, transferring the source code to SoC-FPGAs from different manufacturers is not a straightforward process.

In order to expedite the design process, the proposed methodology places a strong emphasis on the reuse of code. Despite the utilization of FOSS tools that partially automate the design process, the main limitation of the proposed methodology remains the intricate design and the time needed for development. Creating the initial prototype can be a time-consuming task that may require specialized knowledge of hardware design, HDL, synthesis and place-and route tools, as well as setting up an embedded GNU/Linux system for the targeted hardware. However, once the first prototype is available, the addition of new features and the porting of the existing architecture to other SoC-FPGA variants becomes a more practical process, thanks to code reusability, facilitating research on novel algorithms.

The authors reviewed the implementation of a spaceborne GNSS receiver and a GNSS rebroadcaster, showing the flexibility of the proposed architecture. A new proof-of-concept demonstrator was introduced, in the form of a general-purpose multi-frequency and multi-constellation GNSS receiver, serving as a testbed for performance evaluation and for the future implementation of new features. The newly introduced receiver was tested, demonstrating the receiver’s ability to acquire and track GNSS signals and obtain navigation solutions in real-time, multi-frequency, multi-constellation mode. The authors assessed the observables quality, and the precision and accuracy of the navigation solutions using a static and an LEO scenario. The power consumption was estimated to be within the expected range.

Future work involves improving the receiver sensitivity. In line with this, the authors are currently working on the development of a high-sensitivity GNSS receiver using the proposed architecture. The authors are also planning to optimize the tracking process to increase the number of signals that the receiver can simultaneously track. Additionally, there is room for enhancing the accuracy of the observable measurements. The future scope of the present research is the development of a framework architecture and methodology that gradually integrates novel functionalities, allowing comprehensive examination and customization of the complete receiver system. This will streamline the design and deployment of experimental GNSS receivers, incorporating unconventional features specifically for research purposes.

## Data Availability

The functionality of the GNSS-SDR software receiver developed in this research, along with guidelines on its configuration and operation, can be accessed online [12]. The GNSS simulator software used for the observables quality assessment is also available online [75]. The source code for the FPGA is proprietary.

## Sample Availability

The receiver configuration files, the recorded signals used for the receiver sensitivity tests and observables quality tests, and the navigation solutions obtained in the navigation solution accuracy assessment are available on request from the authors.

## Abbreviations

The following abbreviations are used in this manuscript:

GNSS	Global Navigation Satellite System
IC	Integrated Circuit
SoC-FPGA	System-on-Chip Field-Programmable Gate Array
SDR	Software-Defined Radio
LEO	Low Earth Orbit
ASIC	Application Specific Integrated Circuit
FOSS	Free and Open Source Software
CPU	Central Processing Unit
COTS	Commercial Off The Shelf
RFFE	Radio-Frequency Front-End
PC	Personal Computer
IP	Intellectual Property
GPS	Global Positioning System
GPU	Graphics Processing Unit
DSP	Digital Signal Processor
RF	Radio Frequency
PS	Processing System
PL	Programmable Logic
DDR	Double Data Rate
RINEX	Receiver Independent Exchange Format Version
RTCM	Radio Technical Commission for Maritime Services
NMEA	National Marine Electronics Association
DMA	Direct Memory Access
AMBA	Advanced Microcontroller Bus Architecture
AXI4	Advanced eXtensible Interface
OS	Operating System
SD	Secure Digital
AMD	Advanced Micro Devices
MPSoC	Multi-Processor System on Chip
A/D	Analog-to-Digital Converter
PCPS	Parallel Code Phase Search
PRN	Pseudo-Random Noise
CAF	Cross-Ambiguity Function
FFT	Fast Fourier Transform
PVT	Position, Velocity, and Time
FSM	Finite State Machine
GPL	General Public License
PLL	Phase-Locked Loop
DLL	Delay-Locked Loop
LNA	Low-Noise Amplifier
AGC	Automatic Gain Control
SDK	Software Development Kit
HLS	High-Level Synthesis
VHDL	Very-High-Speed Integrated Circuit Hardware Description Language
RTL	Register Transfer Logic
CW	Continuous Wave
SFF	Small Form Factor
RMSE	Root Mean Square Error
Msps	Mega samples per second
ECEF	Earth-Centered, Earth-Fixed
DRMS	Distance Root Mean Square
CEP	Circular Error Probability
SAS	Spherical Accuracy Standard
MRSE	Mean Radial Spherical Error
SEP	Spherical Error Probable

## Appendix A

This section presents the default receiver configuration employed to obtain the experimental results, excluding the test conducted in the LEO scenario. Table A1 shows the configuration of the acquisition block. Table A2 shows the configuration of the tracking blocks. Finally, Table A3 shows the configuration of the PVT block.

Table A1 shows the receiver acquisition parameters: the Doppler max is the maximum Doppler value in the search grid. The Doppler step is the frequency step in the search grid. Self-assistance to acquisition from primary to secondary band is enabled. Therefore, once the primary band is successfully acquired, the receiver predicts the Doppler frequency in the secondary band. Subsequently, it performs acquisition in the secondary band with a reduced Doppler search grid. The threshold parameter refers to the decision threshold from which a signal will be considered present. A downsampling filter is used in the L1/E1 band to reduce the acquisition latency. The downsampling filter is only used in the acquisition.

**Table A1:** Receiver acquisition configuration.

Acquisition GPS L1 C/A	Doppler Max	5000 Hz
	Doppler Step	500 Hz
	Threshold	2.5
	Downsampling Factor	4
Acquisition GPS L5	Doppler Max	500 Hz
	Doppler Step	250 Hz
	Threshold	2.5
	Assistance to acquisition from primary to secondary band	Enabled
Acquisition Galileo E1b/c	Doppler Max	5000 Hz
	Doppler Step	250 Hz
	Threshold	2.5
	Downsampling Factor	4
Acquisition Galileo E5a	Doppler Max	500 Hz
	Doppler Step	125 Hz
	Threshold	2.5
	Assistance to acquisition from primary to secondary band	Enabled

Table A2 shows the receiver tracking configuration parameters: the coherent integration time is set to 20 ms to increase the apparent signal-to-noise ratio. The early–late space chips are the spacing between the Early and Prompt, and between the Prompt and Late correlators, normalized by the chip period. The early-late narrow space chips is the spacing between the Early and Prompt, and between the Prompt and Late correlators, normalized by the chip period after bit synchronization. The very early-late space chips is the spacing between the Very Early and Prompt and between the Prompt and Very Late correlators, normalized by the chip period The very early late space narrow chips is the spacing between the Very Early and Prompt, and between the Prompt and Very Late correlators after removal of the secondary code and extension of the coherent integration time, normalized by the chip period. The PLL filter bandwidth is the bandwidth of the PLL low-pass filter. The PLL filter bandwidth with narrow correlator configuration is the bandwidth of the PLL low-pass filter after removal of the secondary code. The DLL filter bandwidth is the bandwidth of the DLL low-pass filter. Finally, the DLL filter bandwidth with narrow correlator configuration is the bandwidth of the DLL low-pass filter after removal of the secondary code.

Table A3 shows the receiver PVT configuration parameters: the positioning mode is set to single-point positioning. The receiver autonomous integrity monitoring (RAIM) fault detection and exclusion (FDE) is enabled. The ionospheric correction is performed according to the broadcasted ionospheric model. A Saastamoninen troposhperic model is used [79]. The PVT output rate is set to 1 s, and the satellites marked as unhealthy are not used for the computation of the PVT solutions. On top of that, a PVT Kalman filter is used.

More information regarding the GNSS-SDR configurable parameters can be found in [12].

**Table A2:** Receiver tracking configuration.

Tracking GPS L1 C/A	Coherent integration time	20 ms
	Early–Late space chips	0.5
	Early–Late space narrow chips	0.1
	PLL filter bandwidth	35 Hz
	PLL filter bandwidth (narrow correlator configuration)	7.5 Hz
	DLL filter bandwidth	2 Hz
	DLL filter bandwidth (narrow correlator configuration)	0.5 Hz
Tracking GPS L5	Coherent integration time	20 ms
	Early–Late space chips	0.5
	Early–Late space narrow chips	0.1
	PLL filter bandwidth	20 Hz
	PLL filter bandwidth (narrow correlator configuration)	7.5 Hz
	DLL filter bandwidth	1.5 Hz
	DLL filter bandwidth (narrow correlator configuration)	0.5 Hz
Tracking Galileo E1b/c	Coherent integration time	20 ms
	Early–Late space chips	0.25
	Very Early–Late space chips	0.5
	Early–Late space narrow chips	0.15
	Very Early–Late space narrow chips	0.5
	PLL filter bandwidth	15 Hz
	PLL filter bandwidth (narrow correlator configuration)	7.5 Hz
	DLL filter bandwidth	0.75 Hz
	DLL filter bandwidth (narrow correlator configuration)	0.5 Hz
Tracking Galileo E5a	Coherent integration time	20 ms
	Early–Late space chips	0.5
	Early–Late space narrow chips	0.1
	PLL filter bandwidth	20 Hz
	PLL filter bandwidth (narrow correlator configuration)	7.5 Hz
	DLL filter bandwidth	1.5 Hz
	DLL filter bandwidth (narrow correlator configuration)	0.5 Hz

**Table A3:** Receiver PVT configuration.

General	Positioning Mode	Single
	Receiver Autonomous Integrity Monitoring (RAIM) Fault Detection and Exclusion (FDE)	Enabled
	Iono Model	Broadcast
	Trop Model	Saastamoinen
	PVT Output Rate	1 s
	Use unhealthy sats	Disabled
PVT Kalman Filter	Standard deviation of the position estimations	1 m
	Standard deviation of the velocity estimations	0.1 m/s
	Standard deviation of the dynamic system model for position	0.01 m
	Standard deviation of the dynamic system model for velocity	0.001 m/s

## Appendix B

This section provides the receiver configuration that was employed to obtain the experimental results using the LEO scenario. Table A4 shows the configuration of the acquisition block. Table A5 shows the configuration of the tracking blocks. Finally, Table A6 shows the configuration of the PVT block. Self-assistance to acquisition from primary to secondary band is enabled. Therefore, once the primary band is successfully acquired, the receiver predicts the Doppler frequency in the secondary band and performs acquisition in the secondary band with a reduced Doppler frequency space.

**Table A4:** Receiver acquisition configuration when using the LEO scenario.

Acquisition GPS L1 C/A	Doppler Max	50,000 Hz
	Doppler Step	250 Hz
	Threshold	2.5
	Downsampling Factor	4
Acquisition GPS L5	Doppler Max	5000 Hz
	Doppler Step	250 Hz
	Threshold	2.5
	Assistance to acquisition from primary to secondary band	Enabled
Acquisition Galileo E1b/c	Doppler Max	50,000 Hz
	Doppler Step	250 Hz
	Threshold	2.5
	Downsampling Factor	4
Acquisition Galileo E5a	Doppler Max	5000 Hz
	Doppler Step	250 Hz
	Threshold	2.5
	Assistance to acquisition from primary to secondary band	Enabled

**Table A5:** Receiver tracking configuration when using the LEO scenario.

Tracking GPS L1 C/A	Coherent integration time	20 ms
	Early–Late space chips	0.25
	Early–Late space narrow chips	0.15
	PLL filter bandwidth	35 Hz
	PLL filter bandwidth (narrow correlator configuration)	5.0 Hz
	DLL filter bandwidth	2 Hz
	DLL filter bandwidth (narrow correlator configuration)	0.5 Hz
	Enable FLL pull-in	true
	FLL filter bandwidth	10 Hz
	High dynamics	true
Tracking GPS L5	Coherent integration time	5 ms
	Early–Late space chips	0.5
	Early–Late space narrow chips	0.5
	PLL filter bandwidth	50 Hz
	PLL filter bandwidth (narrow correlator configuration)	30 Hz
	DLL filter bandwidth	4 Hz
	DLL filter bandwidth (narrow correlator configuration)	2 Hz
	Enable FLL pull-in	true
	FLL filter bandwidth	2.5 Hz
	High dynamics	true
	Early–Late space chips	0.15
	Very Early–Late space chips	0.5
	Early–Late space narrow chips	0.15
	Very Early–Late space narrow chips	0.5
	PLL filter bandwidth	15 Hz
	PLL filter bandwidth (narrow correlator configuration)	5.0 Hz
	DLL filter bandwidth	0.75 Hz
	DLL filter bandwidth (narrow correlator configuration)	0.5 Hz
	Enable FLL pull-in	true
	FLL filter bandwidth	10 Hz
	High dynamics	true
Tracking Galileo E5a	Coherent integration time	5 ms
	Early–Late space chips	0.5
	Early–Late space narrow chips	0.5
	PLL filter bandwidth	50 Hz
	PLL filter bandwidth (narrow correlator configuration)	30 Hz
	DLL filter bandwidth	4.0 Hz
	DLL filter bandwidth (narrow correlator configuration)	2.0 Hz
	Enable FLL pull-in	true
	FLL filter bandwidth	2.5 Hz
	High dynamics	true

**Table A6:** Receiver PVT configuration when using the LEO scenario.

General	Positioning Mode	Single
	Receiver Autonomous Integrity Monitoring (RAIM) Fault Detection and Exclusion (FDE)	Enabled
	Iono Model	OFF
	Trop Model	OFF
	PVT Output Rate	20 ms
	Use unhealthy sats	Disabled
PVT Kalman Filter	Standard deviation of the position estimations	1 m
	Standard deviation of the velocity estimations	0.1 m/s
	Standard deviation of the dynamic system model for position	0.01 m
	Standard deviation of the dynamic system model for velocity	0.001 m/s
